# Engineering nucleosomes for generating diverse chromatin assemblies

**DOI:** 10.1093/nar/gkab070

**Published:** 2021-02-15

**Authors:** Zenita Adhireksan, Deepti Sharma, Phoi Leng Lee, Qiuye Bao, Sivaraman Padavattan, Wayne K Shum, Gabriela E Davey, Curt A Davey

**Affiliations:** School of Biological Sciences, Nanyang Technological University, 60 Nanyang Drive, 637551, Singapore; NTU Institute of Structural Biology, Nanyang Technological University, 59 Nanyang Drive, 636921, Singapore; School of Biological Sciences, Nanyang Technological University, 60 Nanyang Drive, 637551, Singapore; NTU Institute of Structural Biology, Nanyang Technological University, 59 Nanyang Drive, 636921, Singapore; School of Biological Sciences, Nanyang Technological University, 60 Nanyang Drive, 637551, Singapore; NTU Institute of Structural Biology, Nanyang Technological University, 59 Nanyang Drive, 636921, Singapore; School of Biological Sciences, Nanyang Technological University, 60 Nanyang Drive, 637551, Singapore; School of Biological Sciences, Nanyang Technological University, 60 Nanyang Drive, 637551, Singapore; School of Biological Sciences, Nanyang Technological University, 60 Nanyang Drive, 637551, Singapore; NTU Institute of Structural Biology, Nanyang Technological University, 59 Nanyang Drive, 636921, Singapore; School of Biological Sciences, Nanyang Technological University, 60 Nanyang Drive, 637551, Singapore; NTU Institute of Structural Biology, Nanyang Technological University, 59 Nanyang Drive, 636921, Singapore; School of Biological Sciences, Nanyang Technological University, 60 Nanyang Drive, 637551, Singapore; NTU Institute of Structural Biology, Nanyang Technological University, 59 Nanyang Drive, 636921, Singapore

## Abstract

Structural characterization of chromatin is challenging due to conformational and compositional heterogeneity *in vivo* and dynamic properties that limit achievable resolution *in vitro*. Although the maximum resolution for solving structures of large macromolecular assemblies by electron microscopy has recently undergone profound increases, X-ray crystallographic approaches may still offer advantages for certain systems. One such system is compact chromatin, wherein the crystalline state recapitulates the crowded molecular environment within the nucleus. Here we show that nucleosomal constructs with cohesive-ended DNA can be designed that assemble into different types of circular configurations or continuous fibers extending throughout crystals. We demonstrate the utility of the method for characterizing nucleosome compaction and linker histone binding at near-atomic resolution but also advance its application for tackling further problems in chromatin structural biology and for generating novel types of DNA nanostructures. We provide a library of cohesive-ended DNA fragment expression constructs and a strategy for engineering DNA-based nanomaterials with a seemingly vast potential variety of architectures and histone chemistries.

## INTRODUCTION

The eukaryotic genome is packaged into chromatin by an approximately equal mass of histone proteins, which provide a foundation for modulating gene expression, maintaining genomic stability and regulating DNA transactions in general. Histone octamers composed of H2A, H2B, H3 and H4 core histones assemble variable expanses of ∼160–240 DNA base pairs (bp) into nucleosomes, the repeating units of chromatin (1,2). This chromatin fiber, which can consist of more than one million nucleosomes in tandem for a given chromosome, can in turn be further compacted into higher order structures through the action of a variety of chromatin architectural factors (3–5). These include linker histones, cohesins, condensins, HP1 and CTCF, amongst other factors, which appear to act at different levels of structural hierarchy in condensing nucleosome fiber. In this manner, modulatable structural and chemical features of chromatin—epigenetic factors—collectively dictate cellular differentiation state and activity. And yet our comprehension of how architectural factors operate to maintain the organizational integrity of chromatin lags significantly behind that of the role of other epigenetic regulatory factors in this regard. Moreover, chromatin higher order structure at the level of the organization of interacting/proximal nucleosomes has remained unclear because of conformational and compositional heterogeneity in the cell and dynamic properties that also limit achievable resolution *in vitro*.

Recent advances in imaging and analytical techniques have permitted more detailed insight into chromatin in situ as well as that isolated from natural sources or reconstituted *in vitro* (6–13). This has resulted in the understanding that compact chromatin structure generally consists of irregular interdigitated nucleosome fibers with zigzag features, thereby challenging the long-standing view that higher order structure pertains to folded, helical conformations, referred to as 30 nm fiber (3,4,14). Nonetheless, 30 nm fiber structures may occur in specialized genomic contexts. An improved comprehension of the structural characteristics of nucleosome fibers and the factors that influence local architecture would help clarify the conformational and dynamic behavior of chromatin in the cell.

Recent advances in single particle analysis by cryo-electron microscopy (cryo-EM) have made it possible to obtain near-atomic resolution structures for different nucleosome-chromatin factor assemblies (2,15,16). However, the inherent conformational freedom of these systems tends to significantly limit the resolution that can be achieved. Moreover, the structural heterogeneity of multi-nucleosome systems, such as arrays, imposes further limitations, especially when the interest is in condensed or aggregated states of chromatin under salt conditions similar to those *in vivo*. Crystallographic approaches can potentially provide high resolution insight into compact states of chromatin under near physiological ionic conditions, but they face similar challenges in terms of obtaining an ordered lattice for the system of interest.

Since the first crystal structure of the core region of a nucleosome (in isolation, nucleosome core particle, NCP) was reported in 1997 (17), there have been many high resolution crystal structures solved of NCPs assembled with various DNA sequences and core histone proteins (2,18,19). However, all have in common a blunt-ended (with one recent exception; 20) DNA fragment of length between 145 and 147 bp. In contrast, few crystal structures of nucleosomes having linker DNA (i.e. >147 bp) have been reported, and these entailed mostly initiatives to shed light on chromatin compaction, higher order structure and largely also linker histone binding (21–26). Moreover, the constructs employed were all based on blunt-ended DNA fragments and coincide with lack of DNA continuity in the lattice and generally significant disorder that substantially limited resolution.

We recently reported the first near-atomic resolution structures of nucleosome fibers, which were obtained by crystallizing dinucleosomes engineered with cohesive-ended DNA termini (27). The constructs self-assemble through annealing of the sticky ends, both in the presence and the absence of linker histone, into zigzag fibers that span the length of the crystals and are interdigitated with one another in the lattice. The structures yield detailed insight into nucleosome fiber conformation and packing, showing a critical role played by linker DNA twist/length and a stabilizing function provided by linker histone binding. This allows one to rationalize and extend observations of chromosome architecture and the general heterogeneity of chromatin organization, thereby dispelling some seemingly conflicting reports on cellular chromatin structure.

The search for cohesive-ended DNA constructs, that when assembled into nucleosomes crystallize as continuous fibers, resulted in several generations of designs, many of which yield well diffracting crystals, albeit some with non-continuous (circular) configurations. Here, we describe the engineering approach taken and the variety of nucleosome assembly and packing configurations obtained for the different constructs. Moreover, we provide a library of DNA expression constructs and a framework for further engineering to allow customization for different applications. We envision that the DNA library and methodology could be of use to chromatin structural biologists and biochemists as well as potentially nanotechnologists.

## MATERIALS AND METHODS

### Cloning of DNA fragments

The DNA sequences corresponding to the different nucleosome and dinucleosome assembly constructs (Table 1) are listed in Supplementary Figures S1 (a series), S2 (b/c series) and S3 (d/e/f series). These constructs, in addition to the cohesive-ended 147 ‘bp’ (147s [ID-158575]; 20) and 145 bp Widom-601 sequences (601 [ID-158620], 601L [ID-158572] and 601R [ID-158573]; 19,28), upon which the nucleosome core regions of the a–f series are based, are all available from Addgene (www.addgene.org).

**Table 1.** DNA constructs tested for nucleosome assembly and crystallization

Construct^a^	Generation^b^	Basis^c^	NRL^d^	Shared^e^	Paired^f^	Diffract^g^	Termini^h^
165a	1	NCP	165	–	20	no	PstI
167a	1	NCP	167	–	22	no	PstI
169a	1	NCP	169	–	24	yes	PstI
169ak	1	NCP	169	–	24	yes	KpnI
169an	1	NCP	169		24	yes	KpnI/SacI
171a	1	NCP	171	–	26	no	PstI
173a	1	NCP	173	–	28	no	PstI
175a	1	NCP	175	–	30	no	KpnI
177a	1	NCP	177	–	32	no	PstI
338b	2	169a	169	24	24	yes	EcoRV
343c	3	338b	171.5	29	24	yes	KpnI
345c	3	338b	172.5	29	26	no	KpnI
347c	3	338b	173.5	29	28	no	KpnI
349c	3	338b	174.5	29	30	yes^i^	KpnI
351c	3	338b	175.5	29	32	no	KpnI
353c	3	338b	176.5	29	34	yes	KpnI
357d	4	349c	178.5	29	38	no	KpnI
359d	4	349c	179.5	29	40	no	KpnI
361d	4	349c	180.5	29	42	no	KpnI
351e	5	349c	175.5	31	30	no	KpnI
353e	5	351c	176.5	31	32	yes	KpnI
355e	5	353c	177.5	31	34	yes^i^	KpnI
357e	5	353c	178.5	31	36	no	KpnI
354f	6	353e	177	32	32	yes	KpnI

^a^Construct identity.

^b^Generation number in design process.

^c^Basis upon which construct was designed.

^d^Nucleosome repeat length (bp).

^e^Length of the connecting linker DNA (bp) in the dinucleosome constructs.

^f^Length of the linker DNA (bp) formed by annealing of the termini.

^g^X-ray data set obtained to a resolution of at least 5.5 Å.

^h^Restriction enzyme(s) used to generate termini (4-nucleotide overhangs except for EcoRV, which generates blunt ends).

^i^From Adhireksan *et al.* (27).

The 165–177a series DNA fragments consist of 161–173 bp duplexes with 4-nucleotide overhangs at each 3′ terminus. The palindromic 165a, 167a, 169a, 171a, 173a and 177a sequences were cloned into the pUC19 vector with 8 (165a, 177a) or 16 (167a, 169a, 171a, 173a) copies of two inverted repeat half-sites, based on established doubling strategies (29). This entailed initial BamHI/HindIII-based insertion for the first half-site, followed by sequential concatenation using the HindIII site to couple new insert with vector and BglII/BamHI sites to link the insert fragments (Supplementary Figure S4). The two half-sites each contain a terminal PstI site to yield the 3′ overhangs and a HinfI site to allow generation of the full length fragment via ligation.

The palindromic 169ak and 175a sequences were purchased from GenScript (Piscataway, NJ, USA). The insert for either construct comprises 4 copies of the two inverted repeat half-site pairs inserted into pUC19 vector. The insert is flanked by a BamH1 site on one end and BglII + HindIII sites on the other end to allow further insert doubling if desired (Supplementary Figure S4). The half-sites each contain a terminal KpnI site to yield the 3′ overhangs and a central HinfI site to allow generation of the full length fragment via ligation.

The non-palindromic 169an DNA fragment consists of 165 bp with two non-compatible 4-nucleotide overhangs at each 3′ terminus. The non-palindromic 338b DNA fragment consists of 338 bp with blunt-ended termini. The 169an and 338b DNA fragments were inserted as four (169an) or three (338b) full tandem repeats into pUC19 vector, with KpnI/SacI (169an) or EcoRV (338b) sites flanking each of the repeats (GenScript, Piscataway, NJ, USA). The insert is flanked by a BamH1 site on one end and BglII + HindIII sites on the other end to allow further insert doubling if desired (Supplementary Figure S4).

The non-palindromic 343–353c, 357–361d, 351–357e and 354f dinucleosomal DNA fragments consist of 339–357 bp duplexes with 4-nucleotide overhangs at each 3′ terminus. Three full tandem repeats of the DNA fragments were inserted into pUC19 vector, with KpnI sites flanking each of the repeats (GenScript, Piscataway, NJ, USA). For the 343–353c series, the insert is flanked by a BamH1 site on one end and BglII + HindIII sites on the other end to allow further insert doubling if desired (Supplementary Figure S4). For the d, e and f series, the insert is cloned into the HindIII and EcoRI sites of the vector.

### DNA overexpression and purification

For the palindromic 165–177a and 169ak constructs, the two half-sites were cleaved from pUC19 vector by digestion with PstI (KpnI in the case of 169ak or 175a), followed by FPLC purification using a Resource Q column (GE Healthcare, Chicago, IL, USA) to remove vector. Subsequent to dephosphorylation of the half-sites by calf intestinal alkaline phosphatase (CIP), the DNA was digested by HinfI and subjected to FPLC purification using a Mono Q column (GE Healthcare, Chicago, IL, USA) to remove the spacer DNA fragment. The two half-sites were coupled by ligase treatment and subjected to a final round of Mono Q purification.

For the 169an construct, the DNA fragment was cleaved from the plasmid by digestion with KpnI and SacI, followed by FPLC purification using a Resource Q column (GE Healthcare, Chicago, IL, USA) to remove vector. For the 338b construct, the DNA fragment was cleaved from the plasmid by digestion with EcoRV. Vector was subsequently eliminated by polyethylene glycol precipitation, and trace undigested contaminants were removed using a Mono Q column (GE Healthcare, Chicago, IL, USA).

For the 343–353c, 357–361d, 351–357e and 354f constructs, the DNA fragment was cleaved from the vector by digestion with KpnI, followed by FPLC purification using a Resource Q column (GE Healthcare, Chicago, IL, USA) to remove vector.

### Nucleosomal and linker histone complex assembly

*Homo sapiens* H1.0 and the *G. Gallus* H5 (a.k.a. avian H1.0) were each cloned into the pET-15b vector with an N-terminal hexahistidine tag (EZBiolab Inc., Carmel, IN, USA and GenScript, Piscataway, NJ, USA, respectively). *Homo sapiens* H1x and *H. sapiens* H1.3 were each cloned into the pNIC28-Bsa4 vector with an N-terminal hexahistidine tag (Protein Production Platform, School of Biological Sciences, Nanyang Technological University, Singapore). Linker histones were overexpressed and purified as previously reported for H1.0 (27). N-terminal His-tag was removed with tobacco etch virus (TEV) protease (H1x, H1.3 and H5) or human rhinovirus 3C (HRV3C) protease (H1.0).

The molecular weight of the purified linker histones was determined by mass spectrometry analysis, which confirmed the full length nature of H1x, H1.3 and H1.0. The H5 material obtained, however, is a truncated version of the full length linker histone, whereby the last 43 amino acids are absent from the C-terminus. All nucleosomal constructs and linker histone complexes were assembled with recombinant *H. sapiens* core histones (30,31) and any of the DNA fragments described here, as detailed previously (27; Supplementary Figures S5–S7). Prior to crystallization, assemblies were not purified further subsequent to nucleosomal reconstitution and linker histone purification.

### Crystallization and data collection

Linker histone complexes were prepared for crystallization by mixing linker histone and (di)nucleosome with a slight excess of linker histone (1.2–1.4 linker histone:nucleosome molar stoichiometry). Nucleosomal assemblies with and without linker histone were generally screened for crystallization against divalent metal- (Ca^2+^, Mg^2+^ or Mn^2+^) and spermine-containing buffers, in addition to spermidine buffers or a variety of commercially available and homemade low ionic strength polymer-based screens for systems that did not readily yield well diffracting crystals. Nonetheless, the best diffracting crystals were obtained from Ca^2+^ buffers, with one exception being the 353e construct, for which the highest resolution diffracting crystals were produced with MgCl_2_ buffers, although well diffracting crystals were also obtained in MnCl_2_ and CaCl_2_ buffers. Moreover, the best diffracting crystals overall typically entailed co-crystallization with either the H1x or H1.0 linker histone variant, with the variants being most to least readily/frequently identifiable in electron density maps (allowing incorporation into the model), following the order H1.0, H5, H1x and H1.3.

For yielding the best diffracting crystals, (di)nucleosome with/without linker histone was incubated in buffers consisting of 40–100 mM CaCl_2_ (MgCl_2_ for the 353e dinucleosome), 50 mM KCl and 20 mM Na-acetate [pH 4.5] to give a final total concentration of 4 mg ml^−1^. Crystals were grown at 18°C by the hanging-droplet vapor diffusion method through salting-in via equilibration against a reservoir solution containing 20–50 mM CaCl_2_ (MgCl_2_ for 353e), 25 mM KCl and 10 mM Na-acetate [pH 4.5].

Linker histone assemblies and (di)nucleosome crystals were harvested and stabilized in a buffer consisting of 10–20 mM CaCl_2_ (MgCl_2_ for 353e), 12.5 mM KCl, 10 mM Na-acetate [pH 4.5], 25% 2-methyl-2,4-pentanediol (MPD) and 2% trehalose. For testing X-ray diffraction quality, the MPD concentration of the stabilization buffer was increased gradually up to 65% prior to data set collection, which for some crystal systems yielded a pronounced gain in diffraction quality (note that the 349c and 355e assemblies are described elsewhere; 27). For the 169an, 343c and 353c constructs, the data reported are from linker histone-free crystallization (169an) or co-crystallization with H1.0 (343c) or H1.3 (353c); these crystals were stabilized in 25% MPD. For the 169a, 338b, 353e and 354f constructs, the data reported are from co-crystallization with H1.0 (169a, 353e, 354f) or H1x (338b); these crystals were stabilized in 65% MPD.

Single crystal X-ray diffraction data were recorded, subsequent to mounting stabilized crystals directly into the cryocooling N_2_ gas stream set at −175°C (32), at beam line X06DA of the Swiss Light Source (Paul Scherrer Institute, Villigen, Switzerland) using a Pilatus 2M-F detector and an X-ray wavelength of 1.0 Å. For the 354f crystals, data was collected using a Rigaku FR-X ultra high-intensity microfocus rotating anode X-ray generator with a Pilatus 300 K detector and an X-ray wavelength of 1.54 Å. Data collection statistics are given in Tables 2 and 3 and Supplementary Table S1.

**Table 2.** Data collection and refinement statistics for the 169a and 169an nucleosome and 338b dinucleosome structures

	169a	169an	338b
**Data collection^a^**			
Space group	*P*2_1_	*P*1	*P*2_1_
Cell dimensions			
*a* (Å)	104.80	107.34	103.50
*b* (Å)	102.76	116.54	101.41
*c* (Å)	218.05	117.90	215.67
α/β/γ (°)	90/97.40/90	61.50/82.77/64.23	90/97.48/90
Resolution (Å)^b^	3.20–39.86	3.00–48.07	2.50–97.61
	(3.20–3.37)	(3.00–3.05)	(2.50–2.64)
Unique reflections	75,580	88,754	150,292
*R*_merge_ (%)	10.8 (81.1)	5.6 (88.3)	9.9 (123.7)
*R*_pim_ (%)	8.4 (61.2)	3.7 (72.8)	4.8 (61.7)
*I* / σ*I*	5.3 (1.2)	12.0 (1.0)	9.1 (1.1)
CC$\frac{1}{2}$ (%)	99.3 (55.7)	99.7 (53.9)	99.9 (48.1)
Completeness (%)	99.3 (96.6)	98.6 (93.7)	98.3 (92.9)
Redundancy	3.0 (2.8)	3.5 (3.1)	5.9 (5.5)
**Refinement**			
Resolution (Å)	3.20–39.86	3.00–48.12	2.50–97.61
Reflections used	74 093	86 975	147 213
*R*_work_ / *R*_free_ (%)	20.9 / 26.3	20.9 / 26.1	20.4 / 25.6
No. atoms	27 663	25 911	27 327
Core histone	12 146	12 007	12 711
Linker histone	1647	—	673
DNA	13,846	13 862	13 860
Solvent	24	42	318
*B*-factors (Å^2^)	127	129	85
Core histone	93	94	59
Linker histone	182	—	147
DNA	149	159	106
Solvent	109	94	64
R.m.s. deviations			
Bond lengths (Å)	0.007	0.004	0.009
Bond angles (°)	1.22	1.15	1.43

^a^Single crystal data. Data collection wavelength, 1.0 Å.

^b^Values in parentheses are for the highest-resolution shell.

**Table 3.** Data collection and refinement statistics for the 343c and 353e dinucleosome structures

	343c	353e
**Data collection^a^**		
Space group	*P*2_1_	*P*1
Cell dimensions		
*a* (Å)	107.75	66.21
*b* (Å)	205.90	105.05
*c* (Å)	237.68	171.13
α/β/γ (deg.)	90/97.19/90	86.59/88.95/88.25
Resolution (Å)^b^	3.89–49.30	2.86–49.30
	(3.89–4.10)	(2.86–2.91)
Unique reflections	93,763	103,522
*R*_merge_ (%)	7.1 (175)	8.0 (127)
*R_pim_* (%)	3.2 (77.4)	5.7 (105)
*I* / σ*I*	11.4 (1.1)	7.7 (0.8)
CC$\frac{1}{2}$ (%)	100 (47.8)	99.6 (40.1)
Completeness (%)	99.3 (96.1)	97.4 (82.9)
Redundancy	6.9 (6.7)	3.4 (2.8)
**Refinement**		
Resolution (Å)	3.89–49.30	2.86–49.30
Reflections used	91 832	101 413
*R*_work_ / *R*_free_ (%)	20.1 / 27.0	22.5 / 29.7
No. atoms	53,828	27,785
Core histone	24 548	12 139
Linker histone	1150	1150
DNA	28 130	14 475
Solvent	—	21
*B*-factors (Å^2^)	240	128
Core histone	194	85
Linker histone	335	204
DNA	277	158
Solvent	—	78
R.m.s. deviations		
Bond lengths (Å)	0.005	0.006
Bond angles (°)	1.22	1.44

^a^Single crystal data. Data collection wavelength, 1.0 Å.

^b^Values in parentheses are for the highest-resolution shell.

### Structure solution and refinement

Diffraction data were indexed, integrated, merged, scaled and evaluated with a combination of iMosflm (33,34), XDS (35), autoPROC (36), SCALA (37) and AIMLESS (38) from the CCP4 package (39,40) and in-house data processing pipelines, *go.com* and *go.py*, developed by the Swiss Light Source macromolecular crystallography beamlines (Paul Scherrer Institute, Villigen, Switzerland).

Initial phases for solving structures were obtained by molecular replacement using the program PHASER (41) and MOLREP (42) from the CCP4 package (39,40), with the 2.2 Å resolution crystal structure of NCP composed of the 601L DNA fragment (pdb code: 3UT9) (19) serving as the search model. Linker histone search model elements for molecular replacement included the H1x globular domain NMR structure (pdb code: 2LSO), the avian H5 globular domain from the assembly with a 167 bp nucleosome crystal structure (pdb code: 4QLC; 22) and H1.0 from the 349c nucleosome fiber structure (pdb code: 6LA8; 27).

Atomic refinement and model building were carried out with REFMAC (43) and COOT (44), respectively, from the CCP4 suite (39,40). Molecular replacement using the refined 343c model with 5 bp extensions added to each cohesive terminus and the 353e model as references, respectively, was carried out to confirm the configurations occurring in the 353c and 354f crystals. Structure refinement statistics are given in Tables 2 and 3. Graphic figures were prepared with PyMOL (DeLano Scientific LLC, San Carlos, CA, USA) and CCPmg (45).

## RESULTS

### Engineering nucleosomes for self-assembly

**Figure 1.**
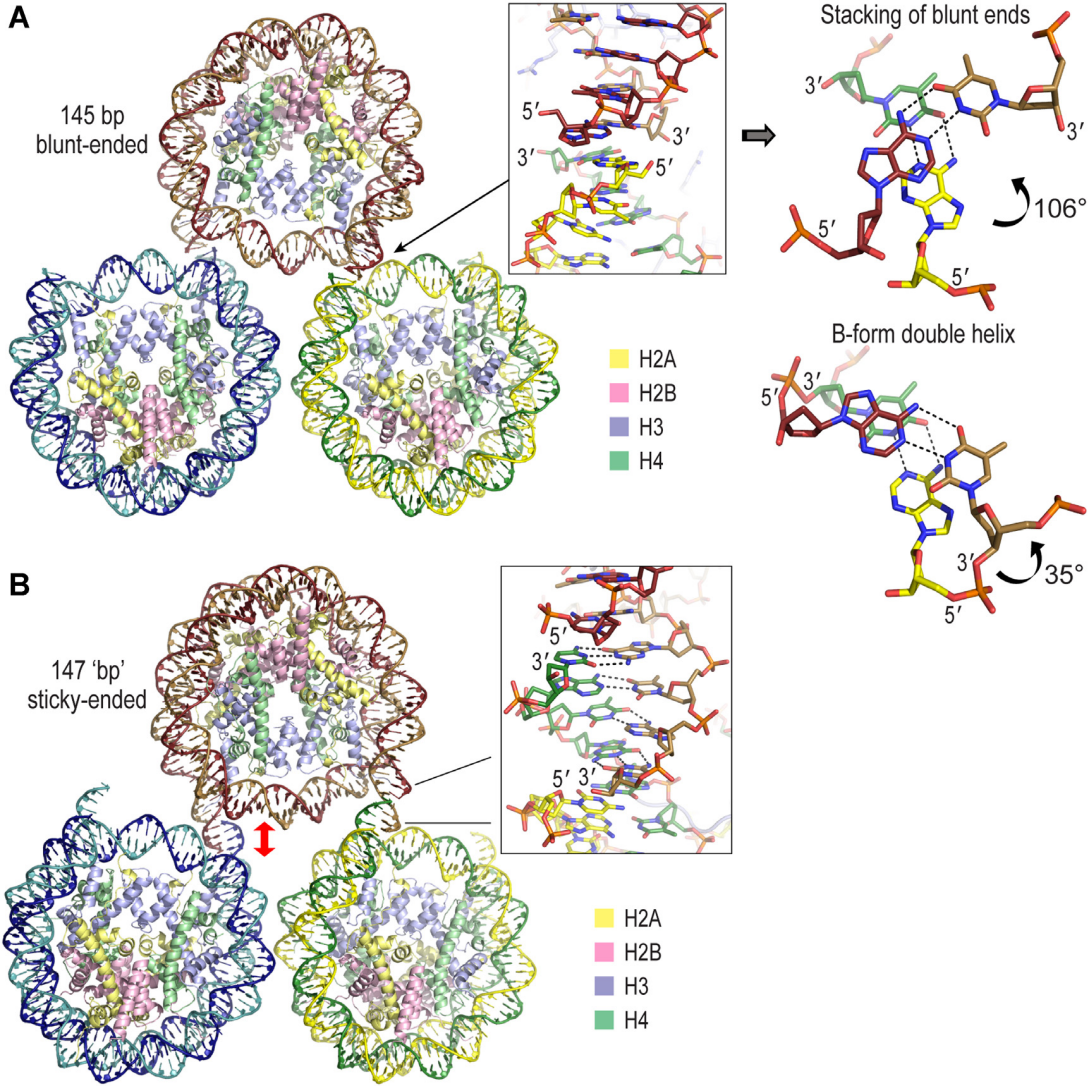
DNA termini interactions in NCP crystal structures. (**A**) DNA termini stacking in blunt-ended NCP crystal structures. The loosely stacking DNA termini make van der Waals contact between the nucleosome cores in the lattice. However, the blunt end junctions coincide with interrupted double helix continuity as the bp step twist relationship between the paired 5′ and 3′ ends is around 3 to 4 times greater than the value (ranging from roughly 106°–147° across blunt-ended NCP crystal structures) that is relevant to B-form DNA (∼35°). The inter-particle DNA stacking shown here is for the structure with the 145 bp 601L sequence (19), which was used as the basis for the core elements of the current nucleosomal constructs. (**B**) Crystal structure of NCP assembled with the cohesive-ended 147s (four nucleotides–143 bp–four nucleotides) DNA fragment (20). The annealing of the cohesive termini imposes an ∼8 Å separation of the particles due to the two additional bp at each junction relative to the 145 bp NCP (red arrow). Note that the 5′ termini of the 145 are dephosphorylated (A; phosphate groups have been added for clarity on the right side scheme), whereas those of the 147s (B) are not.

**Figure 2.**
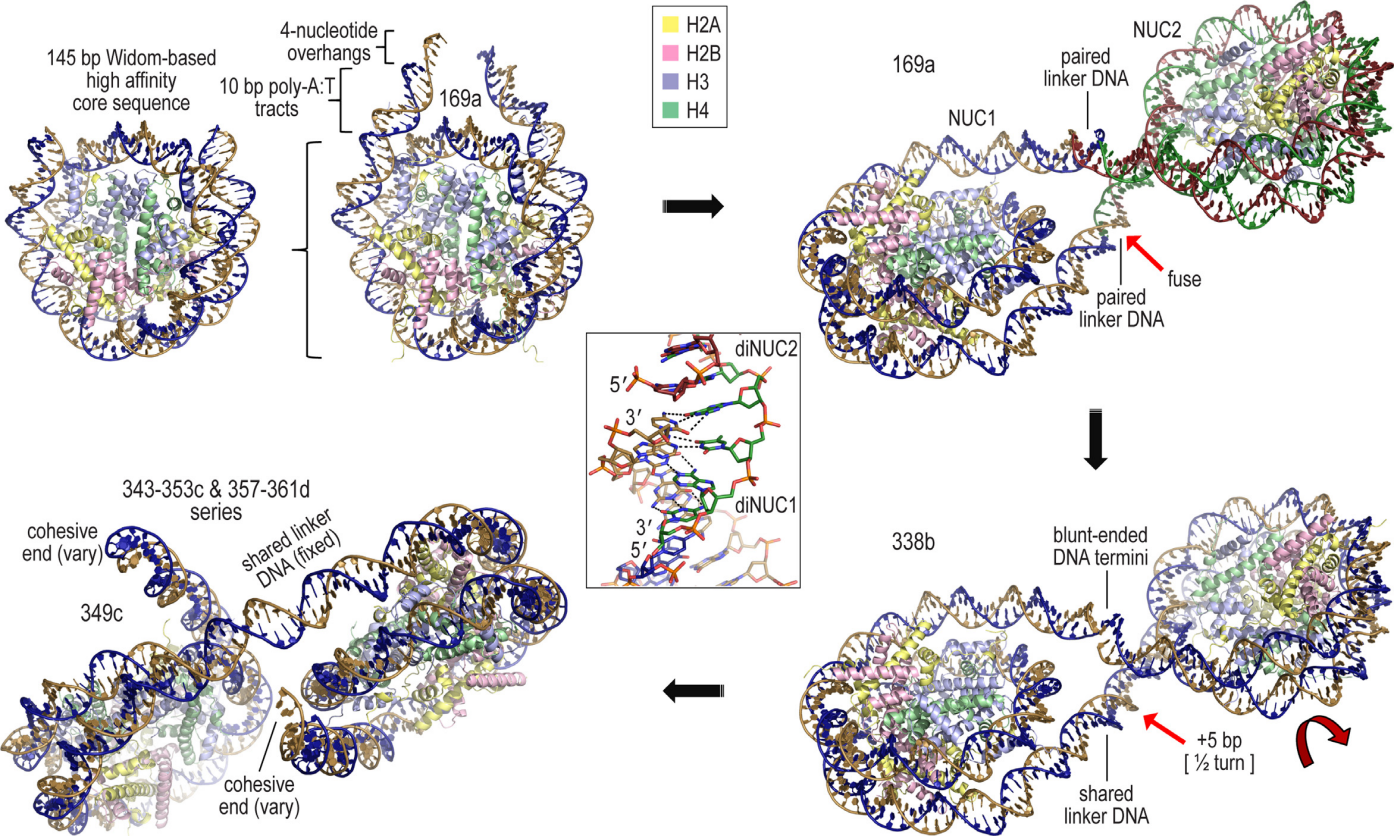
Design process for the nucleosomal constructs. The mono-nucleosome template (top; 169a) was followed by a blunt-ended dinucleosome (338b) and subsequent cohesive-ended dinucleosome assemblies (343–353c and 357–361d series). DNA strands and histone proteins are shown with distinguishing colors (linker histones not shown for clarity; see Figure 4D and Supplementary Figures S8 and S9). The middle inset illustrates the intermolecular annealing of DNA cohesive termini in the lattice (shown for the continuous fiber 349c model, Figure 4D; dashed lines represent hydrogen bonds; 27).

The proclivity of NCPs assembled from 145–147 bp, blunt-ended DNA fragments to form well diffracting crystals is the result of a favored lattice packing of the trim, tuna can-shaped particles, wherein the double helix termini from neighboring NCPs abut in the lattice (Figure 1A; 2, 17–19). The DNA length requirement can be simplified further as high resolution diffracting crystals almost always have an effective double helix length of 147 ‘bp’, whereby occurrences of DNA stretching, each of which extend the double helix relative to the histone octamer register by a single bp, are taken into account (19).

Given that the loosely stacking DNA termini make van der Waals contact between the NCPs in the lattice, one could in principle engineer constructs with cohesive-ended termini (a.k.a. overhangs or sticky ends) that foster inter-particle annealing. To achieve this, the DNA length and twist relationships of the single-stranded termini would need to permit Watson-Crick base pairing and stacking interactions within a favoured nucleosome packing configuration. In B-form DNA, such as in the nucleosome core, bp step twist occupies a relatively narrow range of values of around 35±6° (19). However, in blunt-ended NCP crystal structures, the DNA termini junctions between particles coincide with interrupted double helix continuity as the bp step twist relationship between the paired DNA ends is around 3 to 4 times the value (ranging from roughly 106° to 147°) occurring in B-form DNA (Figure 1A).

We recently reported a cohesive-ended DNA fragment (147s) consisting of a 143 bp core with 4-nucleotide 3′ overhangs at each terminus (20). This construct, assembled into NCP, crystallizes into continuous nucleosome core fibers throughout the lattice via annealing of the sticky ends (Figure 1B). Although the repeats are 147 bp, they have an effective length of 149 bp, since there are two incidences of stretching in the Widom 601-based DNA sequence. The two additional base pairs serve to accommodate the large twist offset between neighboring termini, while imposing a corresponding ∼8 Å separation of particles from the added double helix rise. Importantly, this illustrates a potential dominance of DNA twist relationships—Watson–Crick base pairing of the cohesive termini—over specific nucleosome packing forces.

While the inter-particle annealing 147s construct can facilitate crystallographic characterization of NCPs with diminished stability or elevated dynamics of the DNA termini (e.g. γH2A.X-NCP; 20), our ultimate goal was to engineer constructs that could form continuous nucleosome fibers with a variety of linker DNA lengths (27). By exploring several generations of designs, we discovered an approach that allows acquisition of well diffracting crystals from a seemingly limitless variety of nucleosomal constructs (Figure 2, Tables 1–3; Supplementary Figures S1–S3, S8 and S9; Supplementary Table S1). The initial design trial was based on extrapolation from the NCP lattice, but with cohesive- as opposed to blunt-ended DNA fragments. The prototype mono-nucleosome (first generation, a) design entailed a palindromic 169 ‘bp’ [four nucleotides–165 bp–four nucleotides] DNA fragment (169a; Figure 2, Table 1). The 169a DNA contains a near maximal (consensus) histone octamer-affinity, 145 bp Widom-based (46,47) sequence (601L) (19) and histone octamer-‘repelling’ 10 bp poly-A|T elements in the linker DNA sections, which terminate in single-stranded 3′ TGCA overhangs. The 169a was designed to uphold the offset particle pairing (the two DNA termini of a given particle each pair up with a different particle) displayed by the NCP lattice (Figure 1B). However, due to the angle of exit from the nucleosome core, the linker DNA termini intersect one another, which fosters dimerization of two nucleosomes to form pairs, wherein the self-complementary, 4-nucleotide overhangs from each of the linker DNA termini associate with (canonical) Watson–Crick base pairing (Figure 2). This results in a circular (figure eight) double helix of 338 bp (with two backbone nicks offset by 4 bp at each junction) encompassing the nucleosome dimer, which also corresponds to the asymmetric unit of the crystal.

**Figure 3.**
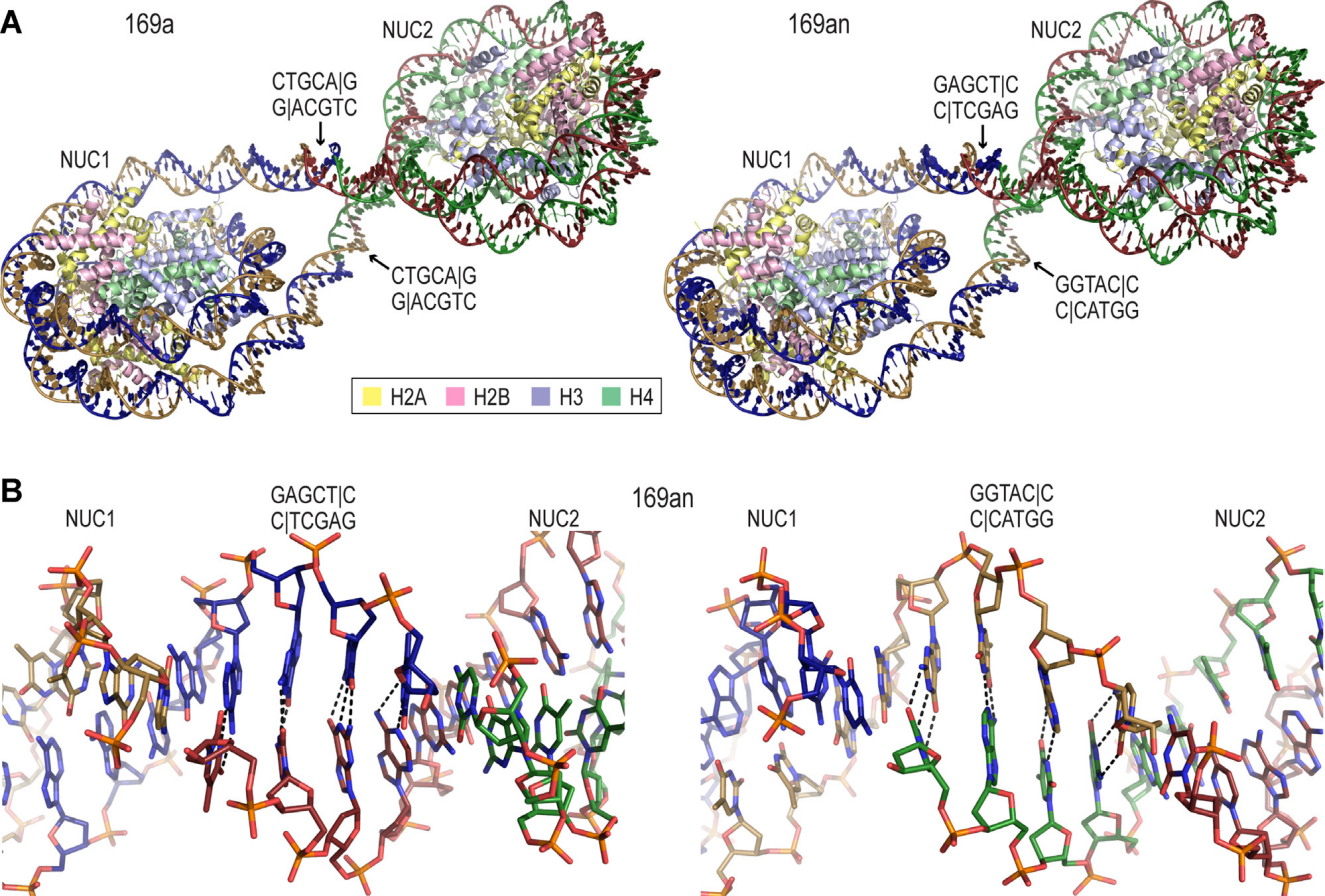
Watson-Crick pairing of distinct cohesive-ended DNA termini in the 169an nucleosome structure. The 169an DNA fragment was engineered with mutually incompatible termini, encompassing a 3′ AGCT overhang (SacI restriction site) at one end and a 3′ GTAC overhang (KpnI restriction site) at the other. Nonetheless, 169an nucleosome dimers compose the lattice, coinciding with the same configuration as for the 169a construct having a 3′ TGCA overhang (PstI restriction site) at either terminus (**A**). The two 169an nucleosomes in the dimer also associate with Watson-Crick pairing of the respective compatible cohesive ends (**B**). DNA strands and histone proteins are shown with distinguishing colors (A, B). Dashed lines represent Watson–Crick hydrogen bonds between the two annealed strands (B).

By co-crystallizing nucleosome assembled with the 169a DNA and different linker histone variants, we obtained near-atomic resolution X-ray diffraction data sets (presented here and unpublished data). Extrapolating from this system, we next wanted to test whether lattice formation could allow discrimination between two different types of cohesive ends, so we designed a non-palindromic version of the 169a DNA, in which the two termini are incompatible. This 169an (n = non-equivalent termini) fragment is engineered with a 3′ GTAC overhang at one end and a 3′ AGCT overhang at the other. Nonetheless, crystals of 169an nucleosome coincide with the same lattice structure as for the 169a nucleosome-linker histone assembly, whereby the two nucleosomes still associate with Watson-Crick pairing of both types of cohesive ends (Figure 3). Importantly, this demonstrates that the pairing of the termini can be DNA sequence-specific, allowing for control of the directionality of the inter-nucleosomal base pairing interactions.

Considering that the 169a/an constructs both yield, via nucleosome pairing, ‘closed’ systems, we surmised that one may be able to obtain alternative lattice configurations by varying the length of the linker DNA sections. We thus designed a series of 169a-type (palindromic) fragments, in which the DNA termini lengths are systematically and symmetrically varied in single base pair steps: yielding the 165a, 167a, 171a, 173a, 175a and 177a constructs (Table 1). However, we did not obtain well diffracting crystals for any of these designs. This suggests that mono-nucleosome constructs that are not predisposed to forming closed dimer pairs likely preside over many interaction degrees of freedom, which disfavors the formation of ordered crystals. Nonetheless, we had only screened the non-169 members of this nucleosome series in the context of assembly with linker histone.

### Cohesive-ended dinucleosomes

In order to depart from the closed nature afforded by the 169a constructs and also reduce the interaction degrees of freedom available to mono-nucleosomes, we wanted to establish whether multi-nucleosome conformation is sufficiently robust/predictable to foster more elaborate engineering approaches. Therefore, we designed a first dinucleosome construct by fusing one of the paired termini in the 169a dimer structure, which yields a (2 × 169) 338 bp DNA. Reconstitution with this blunt-ended DNA fragment (338b) yields two nucleosomes that share a 24 bp linker DNA between them and each have a 12 bp linker DNA terminus. Crystallization yields exceptionally well-ordered crystals, allowing acquisition of a 2.5 Å resolution structure. The 338b lattice and dinucleosome structure are nearly identical to those of the 169a nucleosome pair, with the 338b DNA blunt ends even stacking against one another (Figure 2).

Using this initial dinucleosome (338b) structure as a template, we sought to design a series of fragments that should allow, in principle, formation of open-ended nucleosome arrays in the lattice; that is, with continuity of the double helix from one end to the other in the crystal. As such, we introduced 5 additional bp into the originally 24 bp shared linker DNA section connecting the two nucleosomes. This was intended to create an ∼180° twist offset (B-form DNA repeat≈10.5 bp/turn), which allows the DNA termini from the two different nucleosomes to reside on opposite sides of one another to promote inter-molecular pairing, as opposed to a configuration that favors internal pairing (Figure 2). To foster self-assembly of the dinucleosomes into arrays, we used again cohesive-ended fragments as for the mono-nucleosome systems. Starting with a (338+5) 343 bp fragment, 343c, we also explored systematically and symmetrically lengthening the two DNA termini: yielding the 345c, 347c, 349c, 351c and 353c constructs (Table 1).

### Nucleosome fibers from cohesive-ended dinucleosomes

**Figure 4.**
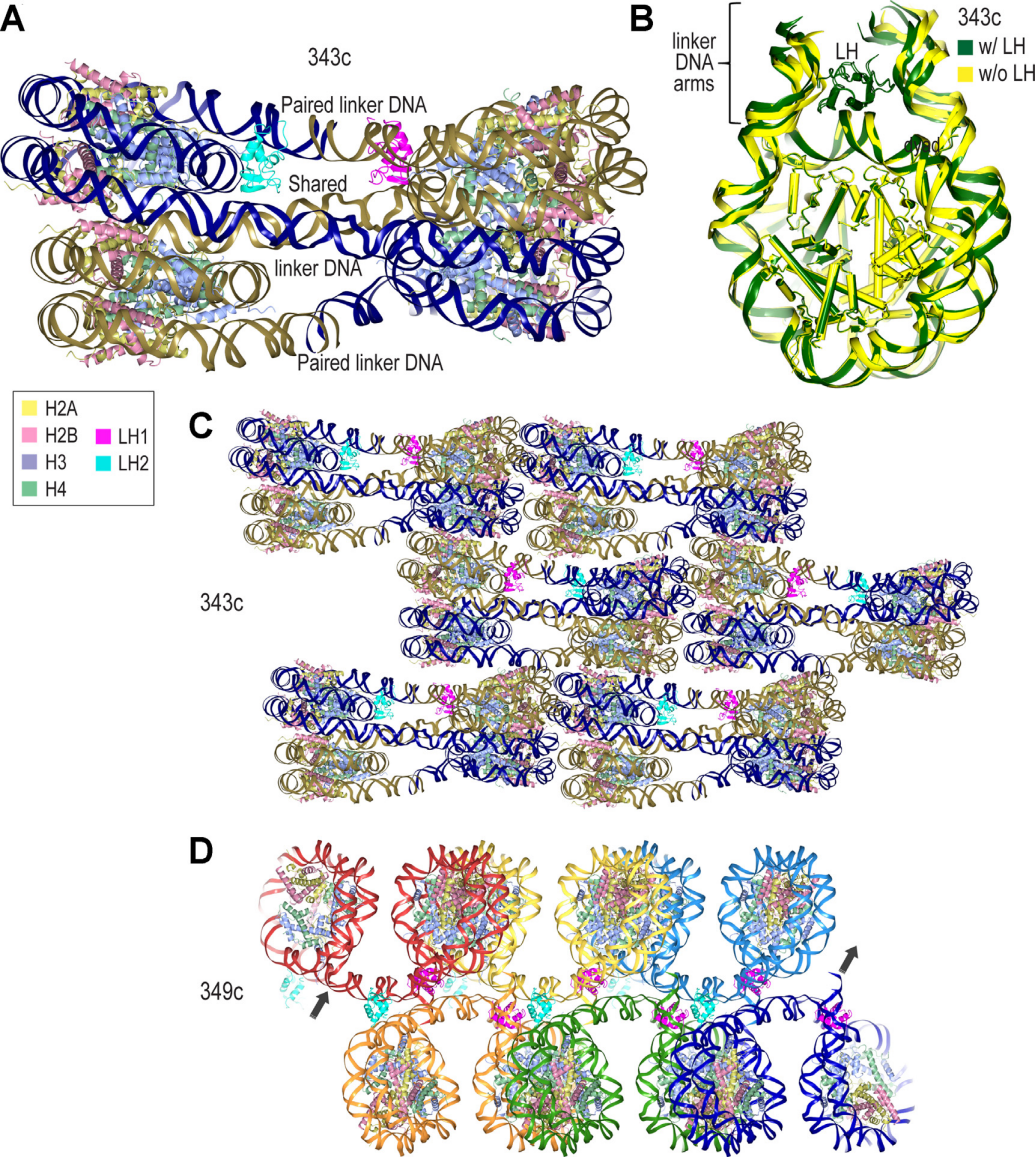
Crystal structures from the 343-353c series. (A–C) The 343c dinucleosome assembled with the H1.0 linker histone variant (LH, magenta/cyan; H1.0 is associated in the on-dyad mode). The cohesive ends from two dinucleosomes (each with distinct colors for the double helix) anneal with each other to form closed tetranucleosome repeating (asymmetric) units (**A**), which stack on top of each other in an offset fashion in the lattice (**C**). The upper and lower nucleosomes (A, B) display conformational differences that coincide with the clarity of electron density associated with linker histone binding, permitting incorporation into the model for only the upper nucleosome layer (**B**; the four unique nucleosomes are superimposed relative to their nucleosome core regions). (**D**) The 349c dinucleosome assembles into continuous fibers (27). The DNA is colored distinctly to distinguish the dinucleosome repeats (asymmetric units).

Well diffracting crystals were obtained for three of the six constructs from the 343c–353c series, which yielded data sets up to 3.4 Å resolution. This includes the smallest construct, 343c, for which two dinucleosomes pair up to generate a closed tetranucleosome system in the crystal (Figure 4). The tetranucleosomes stack on top of each other in an offset fashion, with all nucleosomes situated in the same planar orientation to generate layers that foster face-to-face interactions. The linker DNA connecting the two nucleosomes within the 343c dinucleosome, the ‘shared’ linker, is 29 bp, whereas the one resulting from the termini annealing, the ‘paired’ linker, is 24 bp. This relative length disposition of the two linker sections allows the two dinucleosomes in the tetranucleosome units to stack on each other in an interdigitating fashion, with each of the two nucleosomes in the dinucleosome units residing in separate layers of the lattice. In this configuration, the shorter of the linker sections, the paired linkers, reside on the outside of the tetra-nucleosome.

The largest construct of the 343c-353c series also yielded diffracting crystals, albeit up to only 5.5 Å resolution for the assemblies tested. The 353c lattice is similar to that of 343c (based on a molecular replacement solution), with roughly planar nucleosome stacking. However, the cohesive ends of the 353c, which has 5 bp extensions at either terminus relative to 343c, may not be annealed in the lattice. On the other hand, the 349c construct, with a cohesive-ended linker DNA length nearly intermittent between 343c and 353c, yields a dramatically different self-assembly and packing configuration in the crystal (Figure 4D; 27). The distinct 349c lattice coincides with off-set pairing of dinucleosomes in an open-ended fashion that yields uninterrupted continuity of the DNA double helix from one end of the crystal to the other. The lattice is like that of a fabric woven of nucleosome fibers, which are interdigitated with one another.

Given that the 349c dinucleosomes assemble with their paired linker DNA sections running orthogonal to the nucleosome fiber axis (Figure 4D; 27), we imagined that the same or similar configuration could be supported if the paired linkers were roughly one double helical turn longer. We therefore designed the 357-361d series, where the paired linkers would be 8, 10 or 12 bp longer than those of 349c (Table 1). Nonetheless, from the assemblies tested, well diffracting crystals were not obtained from this family of three constructs.

**Figure 5.**
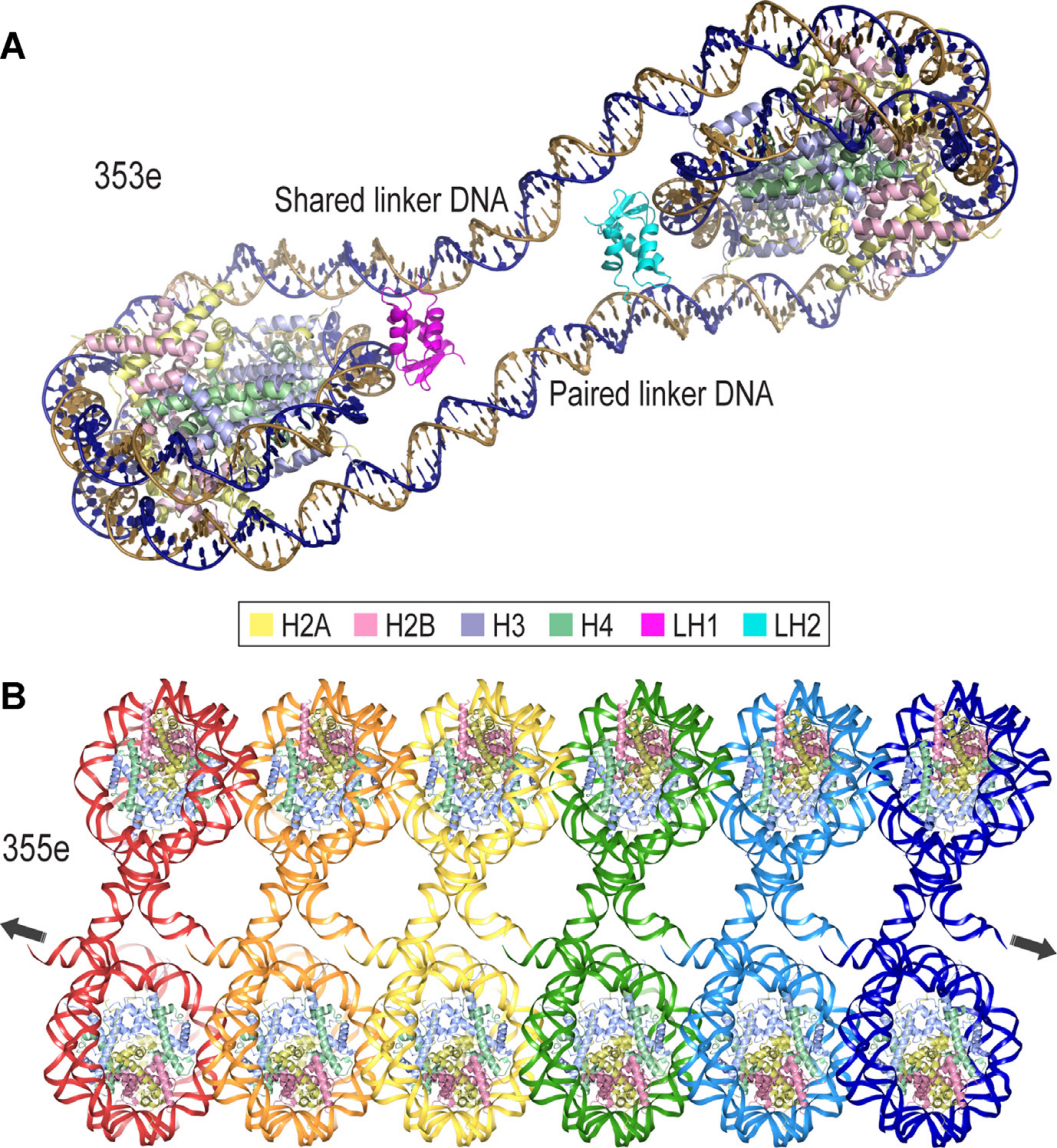
Crystal structures from the 351-357e series. (**A**) The 353e dinucleosome assembled with the H1.0 linker histone variant (LH, magenta/cyan; H1.0 is associated in the on-dyad mode). The cohesive ends from a single dinucleosome (the two DNA strands colored distinctly) anneal with each other to form closed dinucleosome repeating (asymmetric) units. (**B**) The 355e dinucleosome assembles into continuous fibers (27). The DNA is colored distinctly to distinguish the dinucleosome repeats (asymmetric units).

We next tested a series, 351e–357e, where 2 bp were introduced into the shared linker DNA, giving 31 bp relative to the 29 bp for the c and d generations (Table 1). Out of the four constructs, two yielded well diffracting crystals. 353e gives a closed monomeric system, in which the two cohesive termini have paired with each other within the dinucleosome (Figure 5A). On the other hand, the 355e construct gives a second type of continuous fiber system, which is distinct from that of 349c, both in terms of fiber configuration and interdigitation (Figure 5B; 27). We tested a final construct, most similar to 353e, but with the addition of a single bp to the shared linker. Upon annealing in the lattice, this gives the 354f dinucleosome symmetric lengths of 32 bp for both the shared and paired linkers (Table 1). The 354f structure is nonetheless a closed dinucleosome system, very similar to that of 353e (based on a molecular replacement solution).

## DISCUSSION

By introducing DNA annealing as a site-specific and directional driving force, cohesive-ended nucleosomes can promote self-assembly into well-ordered lattices with a variety of possible configurations. The sensitivity of the nucleosome interaction and packing outcome towards the length and character of the DNA fragment—in particular, the linker DNA or nucleosome repeat length—speaks not only for the design potential of the approach to generate diverse assemblies, but also rationalizes the heterogeneity of chromatin structure in the cell (6,9), where individual nucleosomes differ widely in both DNA and histone composition (27). Nonetheless, we have just scratched the surface of the vast condition and design space available, as we have so far tested this approach for 24 mononucleosome and dinucleosome constructs in the context of either no additional chromatin factors or in the presence of linker histone. A detailed analysis of linker histone structure and nucleosome binding will be presented elsewhere. Here, we make the library that we currently have available as there may be unforeseen applications for any particular construct or type thereof. For instance, beyond crystallographic approaches, the constructs may be of use for annealing-based end-labelling in biochemical or biophysical studies, DNA repair investigations or electron microscopy and other 2D/3D imaging studies.

By limiting the accessible intermolecular interaction landscape relative to mononucleosomes, the dinucleosome designs are more likely to favor ordered crystal formation based on what we have tested so far. However, while the presence of linker histone is not observed to have a substantial effect on nucleosome configuration in the crystals (here and 27), we should note that the inclusion of other types of chromatin factors could potentially influence the self-assembly process in a distinct and decisive fashion. In any case, within a dinucleosome platform, co-varying the linker DNA length between the two nucleosomes and at their cohesive termini is a fruitful design principle. This yielded a variety of well diffracting crystals and configurations, including open-fibrous, and closed-circular, systems.

DNA nanotechnology has relied on the special properties of single-stranded nucleic acid molecules, which in the presence of complementary strands are prone to form ‘predictable’ double helical species by following simple base pairing and stacking rules, as opposed to folding into complex (irregular) tertiary structures (48–51). This has fostered the field of DNA origami, where typically hundreds of short oligonucleotides (staple strands) are designed to anneal with long (scaffold) oligonucleotides. In this manner, a great variety of different 2D and 3D shapes, objects and lattices have been fabricated, with applications spanning the sciences and engineering, such as photonics, electronics and synthetic enzymology, to name a few. Indeed, the technique has been used to measure nucleosome-nucleosome interaction potential (52). Moreover, the crystals that can be generated by this approach have use not only as novel biomolecules but also allow for the formulation of precise periodic nanoparticles and quantum dots. In this regard, we suggest that a chromatin- or nucleosome-based platform could expand both the structural and chemical space (histone variants, mutants or synthetic chemistries) available to DNA nanotechnology.

**Figure 6.**
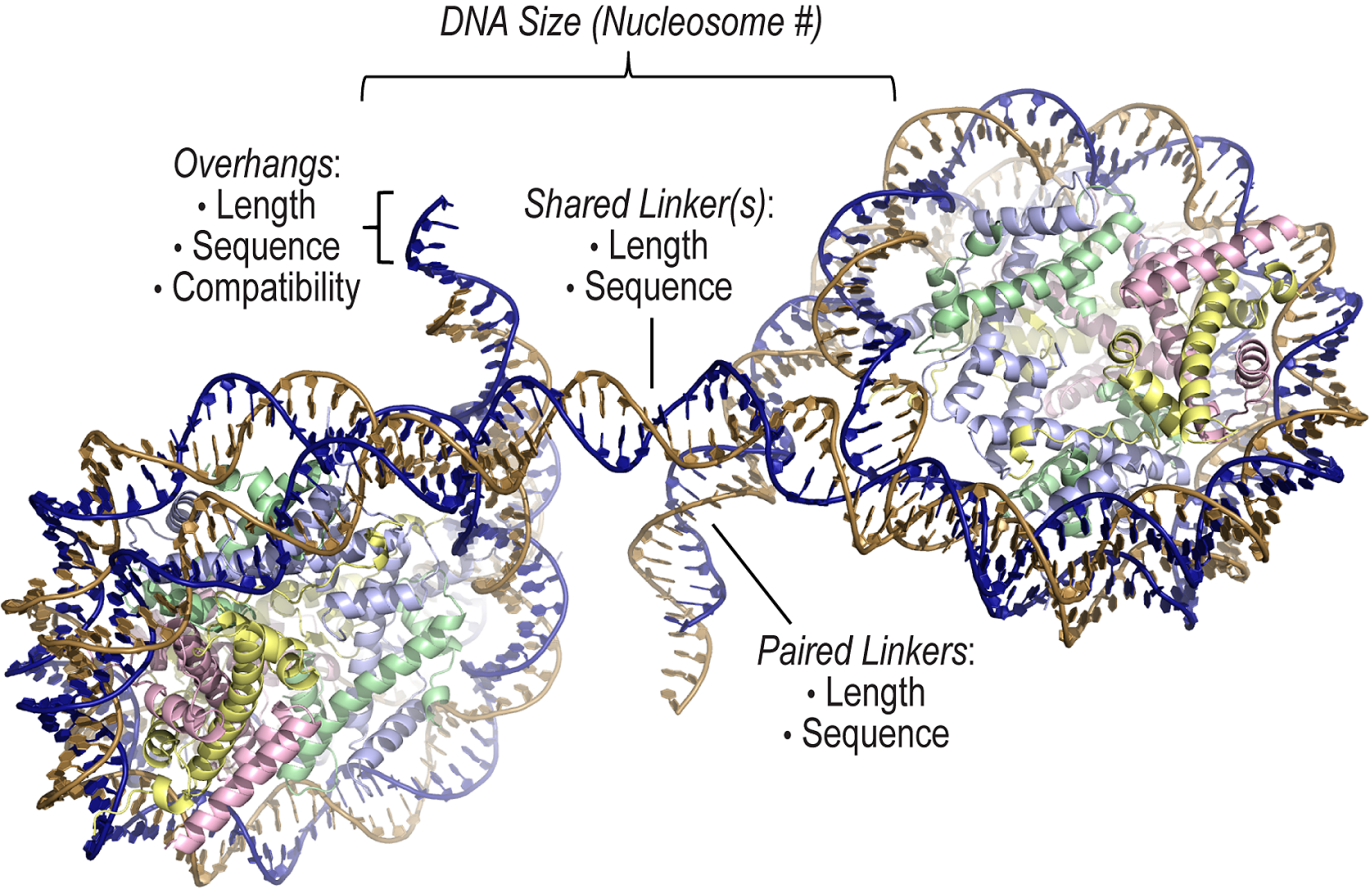
Scheme for the design of cohesive-ended nucleosomal constructs.

Depending on the application sought after, there may be need to engineer constructs that differ with respect to what is presented here. We envision six key variables in the DNA fragment design process (Figure 6): (i) number of nucleosomes (total DNA size), (ii) length of the single-stranded overhangs, (iii) sequence of the overhangs, (iv) compatibility of the overhangs, (v) lengths of shared and paired linker DNA and (vi) sequence of the linker DNA sections. While we do not anticipate a significant role played by the DNA sequence in the nucleosome core regions (as long as singular positioning is maintained) given the conservation of its overall structure across DNA sequence space, this will depend on the intended application, and it may be important to keep in mind that the occurrence of double helix stretching is DNA sequence dependent (19). Thus, for instance Widom 601 sequences display stretching at two locations in the nucleosome core, which correspondingly increases the linker DNA length by one bp at each terminus, yielding 145 as opposed to 147 bp nucleosome cores (in both the crystalline [19,27,28] and solution [53] states). Regarding the linker DNA sections, both shared and paired, their lengths will have a dominant effect on configuration and packing, but the sequence character could also be important as it effects twist and flexibility characteristics (27). Given that annealing of the cohesive termini can translate to a dominant driving force in the assembly process, the length and sequence of the single-stranded overhangs could be modulated to optimize the strength of this driver for a particular construct and application. Moreover, the termini can be designed to be either compatible with one another or not, in the event that one desires orientational, or terminus-specific, discrimination in the assembly process. Indeed, incompatible termini could be introduced to suppress intramolecular annealing (as occurs in the 353e and 354f crystals). Although we have focused largely on dinucleosome constructs here, future designs could impose distinct constraints on the interaction landscape, for instance with tri- or tetra-nucleosomal systems.

By fostering ordered lattice formation, the nucleosome engineering platform presented here could be exploited in future chromatin biology applications beyond compaction, linker histone binding and histone variant/modification studies, such as for the structural characterization of nucleosome fibers composed of *bona fide* genomic sequences or assembled with various (non-histone) chromatin factors. Additionally, the annealed cohesive termini in the lattice could allow for synthesis of plasmid- and chromosome-length DNA fragments by employing chemical ligation techniques (54,55). Nevertheless, only the surface has been scratched so far and yet the apparent productive capacity underscores the potential of the method for applications spanning biology and nanotechnology. Indeed, further exploration is certain to uncover new principles for more elaborate and predictive designs.

## DATA AVAILABILITY

DNA expression constructs are available from Addgene (www.addgene.org). Atomic coordinates and structure factors for the 169a, 169an, 338b, 343c and 353e models have been deposited in the Protein Data Bank under accession codes 6LAB, 6LER, 6L9Z, 6LA2 and 7COW, respectively.

## Supplementary Material

gkab070_Supplemental_File

## Notes

Present address: Qiuye Bao, Programme in Stem Cell, Regenerative Medicine and Ageing, A*STAR Institute of Molecular and Cell Biology, Singapore 138673.

Present address: Sivaraman Padavattan, Department of Biophysics, National Institute of Mental Health and Neurosciences, Bangalore 560029, India.
