# Supplementary material for: Engineering nucleosomes for generating diverse chromatin assemblies
Source: Nucleic Acids Res. 2021 Feb 15;49(9):e52. doi: 10.1093/nar/gkab070 (PMC8136823; doi:10.1093/nar/gkab070)
Supplement: gkab070_Supplemental_File [file gkab070_supplemental_file.pdf]

Engineering Nucleosomes for Generating Diverse Chromatin Assemblies

Zenita Adhireksan, Deepti Sharma, Phoi Leng Lee, Qiuye Bao,

Sivaraman Padavattan, Wayne K. Shum, Gabriela E. Davey & Curt A. Davey

SUPPLEMENTARY DATA

CONTENTS

9 Figures: pages 2-14

1 Table: page 15

165a
GTTTTTTTTTTCCAATCCCGGTGCCGAGGCCGCTCAATTGGTCGTAGACAGCTCTAGCACCGCTTAAACGCACGTA
CGGA(**A**)TCCGTACGTGCGTTTAAGCGGTGCTAGAGCTGTCTACGACCAATTGAGCGGCCTCGGCACCGGGATTG
GAAAAAAAAAACTGCA

167a
GTTTTTTTTTTCACAATCCCGGTGCCGAGGCCGCTCAATTGGTCGTAGACAGCTCTAGCACCGCTTAAACGCACGT
ACGGA(**A**)TCCGTACGTGCGTTTAAGCGGTGCTAGAGCTGTCTACGACCAATTGAGCGGCCTCGGCACCGGGATT
GTGAAAAAAAAAACTGCA

169a
GCTTTTTTTTTTCACAATCCCGGTGCCGAGGCCGCTCAATTGGTCGTAGACAGCTCTAGCACCGCTTAAACGCACG
TACGGA(**A**)TCCGTACGTGCGTTTAAGCGGTGCTAGAGCTGTCTACGACCAATTGAGCGGCCTCGGCACCGGGATT
GTGAAAAAAAAAAGCTGCA

169ak {KpnI}
CCTTTTTTTTTTCACAATCCCGGTGCCGAGGCCGCTCAATTGGTCGTAGACAGCTCTAGCACCGCTTAAACGCACG
TACGGA(**A**)TCCGTACGTGCGTTTAAGCGGTGCTAGAGCTGTCTACGACCAATTGAGCGGCCTCGGCACCGGGATT
GTGAAAAAAAAAAGGGTAC

169an {KpnI/SacI}
Strand 1
CCAAAAAAAAAACAGCATCCCGGTGCCGAGGCCGCTCAATTGGTCGTAGACAGCTCTAGCACCGCTTAAACGCAC
GTACGCGCTGTCTACCGCGTTTTAACCGCCACTAGAAGCGCTTACTAGTCTCCAGGCACGTGTGAGACCGGCACA
TGCAAAAAAAAAACGAGCT
Strand 2
CGTTTTTTTTTTGCATGTGCCGGTCTCACACGTGCCTGGAGACTAGTAAGCGCTTCTAGTGGCGGTTAAAACGCGG
TAGACAGCGCGTACGTGCGTTTAAGCGGTGCTAGAGCTGTCTACGACCAATTGAGCGGCCTCGGCACCGGGATG
CTGTTTTTTTTTTGGGTAC

171a
GCCTTTTTTTTTTCACAATCCCGGTGCCGAGGCCGCTCAATTGGTCGTAGACAGCTCTAGCACCGCTTAAACGCAC
GTACGGA(**A**)TCCGTACGTGCGTTTAAGCGGTGCTAGAGCTGTCTACGACCAATTGAGCGGCCTCGGCACCGGGA
TTGTGAAAAAAAAAAGGCTGCA

173a
GTGCTTTTTTTTTTCACAATCCCGGTGCCGAGGCCGCTCAATTGGTCGTAGACAGCTCTAGCACCGCTTAAACGCA
CGTACGGA(**A**)TCCGTACGTGCGTTTAAGCGGTGCTAGAGCTGTCTACGACCAATTGAGCGGCCTCGGCACCGGG
ATTGTGAAAAAAAAAAGCACTGCA

175a {KpnI}
CCTGCTTTTTTTTTTCACAATCCCGGTGCCGAGGCCGCTCAATTGGTCGTAGACAGCTCTAGCACCGCTTAAACGC
ACGTACGGA(**A**)TCCGTACGTGCGTTTAAGCGGTGCTAGAGCTGTCTACGACCAATTGAGCGGCCTCGGCACCGG
GATTGTGAAAAAAAAAAGCAGGGTAC

177a
GACTGCTTTTTTTTTTCACAATCCCGGTGCCGAGGCCGCTCAATTGGTCGTAGACAGCTCTAGCACCGCTTAAACG
CACGTACGGA(**A**)TCCGTACGTGCGTTTAAGCGGTGCTAGAGCTGTCTACGACCAATTGAGCGGCCTCGGCACCG
GGATTGTGAAAAAAAAAAGCAGTCTGCA

Supplementary Figure S1. DNA sequences of the mono-nucleosome constructs (165-177a). For the palindromic sequences, the central nucleotide (bold) in parentheses is A

in one strand and T in the complementary strand. The restriction enzyme(s) used to generate the cohesive ends is PstI, unless otherwise indicated after the construct name.

338b

Strand 1

ATCGCGGAAAAAAAAACGCATCCCGGTGCCGAGGCCGCTCAATTGGTCGTAGACAGCTCTAGCACCGCTTAAACGC
ACGTACGCGCTGTCTACCGCGTTTTAACCGCCACTAGAAGCGCTTACTAGTCTCCAGGCACGTGTGAGACCGGCA
CATGAAAAAAAAAAAGCAGGAGCGCAAAAAAAAAACGCATCCCGGTGCCGAGGCCGCTCAATTGGTCGTAGACA
GCTCTAGCACCGCTTAAACGCACGTACGCGCTGTCTACCGCGTTTTAACCGCCACTAGAAGCGCTTACTAGTCTCC
AGGCACGTGTGAGACCGGCACATGAAAAAAAAACCGCGAT

Strand 2

ATCGCGGTTTTTTTTCATGTGCCGGTCTCACACGTGCCTGGAGACTAGTAAGCGCTTCTAGTGGCGGTTAAAACGC
GGTAGACAGCGCGTACGTGCGTTTAAGCGGTGCTAGAGCTGTCTACGACCAATTGAGCGGCCTCGGCACCGGGA
TGCGTTTTTTTTTTGCGCTCCTGCTTTTTTTTTTCATGTGCCGGTCTCACACGTGCCTGGAGACTAGTAAGCGCTTCT
AGTGGCGGTTAAAACGCGGTAGACAGCGCGTACGTGCGTTTAAGCGGTGCTAGAGCTGTCTACGACCAATTGAG
CGGCCTCGGCACCGGGATGCGTTTTTTTTCCGCGAT

343c

Strand 1

CGCTGAAAAAAAAACGCATCCCGGTGCCGAGGCCGCTCAATTGGTCGTAGACAGCTCTAGCACCGCTTAAACGCAC
GTACGCGCTGTCTACCGCGTTTTAACCGCCACTAGAAGCGCTTACTAGTCTCCAGGCACGTGTGAGACCGGCACA
TGAAAAAAAAAAGCATGCTCGAGTATGAAAAAAAAAACGCATCCCGGTGCCGAGGCCGCTCAATTGGTCGTAGA
CAGCTCTAGCACCGCTTAAACGCACGTACGCGCTGTCTACCGCGTTTTAACCGCCACTAGAAGCGCTTACTAGTCT
CCAGGCACGTGTGAGACCGGCACATGAAAAAAAAACAGCGGTAC

Strand 2

CGCTGTTTTTTTTCATGTGCCGGTCTCACACGTGCCTGGAGACTAGTAAGCGCTTCTAGTGGCGGTTAAAACGCGG
TAGACAGCGCGTACGTGCGTTTAAGCGGTGCTAGAGCTGTCTACGACCAATTGAGCGGCCTCGGCACCGGGATG
CGTTTTTTTTTTCATACTCGAGCATGCTTTTTTTTTTCATGTGCCGGTCTCACACGTGCCTGGAGACTAGTAAGCGCT
TCTAGTGGCGGTTAAAACGCGGTAGACAGCGCGTACGTGCGTTTAAGCGGTGCTAGAGCTGTCTACGACCAATT
GAGCGGCCTCGGCACCGGGATGCGTTTTTTTTCAGCGGTAC

345c

Strand 1

CGCTGAAAAAAAAAACGCATCCCGGTGCCGAGGCCGCTCAATTGGTCGTAGACAGCTCTAGCACCGCTTAAACGCA
CGTACGCGCTGTCTACCGCGTTTTAACCGCCACTAGAAGCGCTTACTAGTCTCCAGGCACGTGTGAGACCGGCAC
ATGAAAAAAAAAAAGCATGCTCGAGTATGAAAAAAAAAAACGCATCCCGGTGCCGAGGCCGCTCAATTGGTCGTAG
ACAGCTCTAGCACCGCTTAAACGCACGTACGCGCTGTCTACCGCGTTTTAACCGCCACTAGAAGCGCTTACTAGTC
TCCAGGCACGTGTGAGACCGGCACATGAAAAAAAAAACAGCGGTAC

Strand 2

CGCTGTTTTTTTTTCATGTGCCGGTCTCACACGTGCCTGGAGACTAGTAAGCGCTTCTAGTGGCGGTTAAAACGCG
GTAGACAGCGCGTACGTGCGTTTAAGCGGTGCTAGAGCTGTCTACGACCAATTGAGCGGCCTCGGCACCGGGAT
GCGTTTTTTTTTTTCATACTCGAGCATGCTTTTTTTTTTTCATGTGCCGGTCTCACACGTGCCTGGAGACTAGTAAGCG
CTTCTAGTGGCGGTTAAAACGCGGTAGACAGCGCGTACGTGCGTTTAAGCGGTGCTAGAGCTGTCTACGACCAAT
TGAGCGGCCTCGGCACCGGGATGCGTTTTTTTTTCAGCGGTAC

347c

Strand 1

CGCTGAAAAAAAAAAACGCATCCCGGTGCCGAGGCCGCTCAATTGGTCGTAGACAGCTCTAGCACCGCTTAAACGC
ACGTACGCGCTGTCTACCGCGTTTTAACCGCCACTAGAAGCGCTTACTAGTCTCCAGGCACGTGTGAGACCGGCA
CATGAAAAAAAAAAAGCATGCTCGAGTATGAAAAAAAAAAACGCATCCCGGTGCCGAGGCCGCTCAATTGGTCGTA
GACAGCTCTAGCACCGCTTAAACGCACGTACGCGCTGTCTACCGCGTTTTAACCGCCACTAGAAGCGCTTACTAGT
CTCCAGGCACGTGTGAGACCGGCACATGAAAAAAAAAAACAGCGGTAC

Strand 2

CGCTGTTTTTTTTTTTCATGTGCCGGTCTCACACGTGCCTGGAGACTAGTAAGCGCTTCTAGTGGCGGTTAAAACGC
GGTAGACAGCGCGTACGTGCGTTTAAGCGGTGCTAGAGCTGTCTACGACCAATTGAGCGGCCTCGGCACCGGGA
TGCGTTTTTTTTTTTCATACTCGAGCATGCTTTTTTTTTTTCATGTGCCGGTCTCACACGTGCCTGGAGACTAGTAAGC
GCTTCTAGTGGCGGTTAAAACGCGGTAGACAGCGCGTACGTGCGTTTAAGCGGTGCTAGAGCTGTCTACGACCA
ATTGAGCGGCCTCGGCACCGGGATGCGTTTTTTTTTTTCAGCGGTAC

349c

Strand 1

CGCTGGAAAAAAAAAACGCATCCCGGTGCCGAGGCCGCTCAATTGGTCGTAGACAGCTCTAGCACCGCTTAAAC
GCACGTACGCGCTGTCTACCGCGTTTTAACCGCCACTAGAAGCGCTTACTAGTCTCCAGGCACGTGTGAGACCGG
CACATGAAAAAAAAAAGCATGCTCGAGTATGAAAAAAAAAACGCATCCCGGTGCCGAGGCCGCTCAATTGGTCG
TAGACAGCTCTAGCACCGCTTAAACGCACGTACGCGCTGTCTACCGCGTTTTAACCGCCACTAGAAGCGCTTACTA
GTCTCCAGGCACGTGTGAGACCGGCACATGAAAAAAAAAACCAGCGGTAC

Strand 2

CGCTGGTTTTTTTTTTCATGTGCCGGTCTCACACGTGCCTGGAGACTAGTAAGCGCTTCTAGTGGCGGTTAAAACG
CGGTAGACAGCGCGTACGTGCGTTTAAGCGGTGCTAGAGCTGTCTACGACCAATTGAGCGGCCTCGGCACCGGG
ATGCGTTTTTTTTTTCATACTCGAGCATGCTTTTTTTTTTCATGTGCCGGTCTCACACGTGCCTGGAGACTAGTAAG
CGCTTCTAGTGGCGGTTAAAACGCGGTAGACAGCGCGTACGTGCGTTTAAGCGGTGCTAGAGCTGTCTACGACC
AATTGAGCGGCCTCGGCACCGGGATGCGTTTTTTTTTTCCAGCGGTAC

351c

Strand 1

CGCTGCGAAAAAAAAAACGCATCCCGGTGCCGAGGCCGCTCAATTGGTCGTAGACAGCTCTAGCACCGCTTAAAC
GCACGTACGCGCTGTCTACCGCGTTTTAACCGCCACTAGAAGCGCTTACTAGTCTCCAGGCACGTGTGAGACCGG
CACATGAAAAAAAAAAGCATGCTCGAGTATGAAAAAAAAAACGCATCCCGGTGCCGAGGCCGCTCAATTGGTCG
TAGACAGCTCTAGCACCGCTTAAACGCACGTACGCGCTGTCTACCGCGTTTTAACCGCCACTAGAAGCGCTTACTA
GTCTCCAGGCACGTGTGAGACCGGCACATGAAAAAAAAAACGCAGCGGTAC

Strand 2

CGCTGCGTTTTTTTTTTCATGTGCCGGTCTCACACGTGCCTGGAGACTAGTAAGCGCTTCTAGTGGCGGTTAAAAC
GCGGTAGACAGCGCGTACGTGCGTTTAAGCGGTGCTAGAGCTGTCTACGACCAATTGAGCGGCCTCGGCACCGG
GATGCGTTTTTTTTTTCATACTCGAGCATGCTTTTTTTTTTCATGTGCCGGTCTCACACGTGCCTGGAGACTAGTAA
GCGCTTCTAGTGGCGGTTAAAACGCGGTAGACAGCGCGTACGTGCGTTTAAGCGGTGCTAGAGCTGTCTACGAC
CAATTGAGCGGCCTCGGCACCGGGATGCGTTTTTTTTTTCGCAGCGGTAC

353c

Strand 1

CGCTGACGAAAAAAAAAACGCATCCCGGTGCCGAGGCCGCTCAATTGGTCGTAGACAGCTCTAGCACCGCTTAAA
CGCACGTACGCGCTGTCTACCGCGTTTTAACCGCCACTAGAAGCGCTTACTAGTCTCCAGGCACGTGTGAGACCG
GCACATGAAAAAAAAAAGCATGCTCGAGTATGAAAAAAAAAACGCATCCCGGTGCCGAGGCCGCTCAATTGGTC
GTAGACAGCTCTAGCACCGCTTAAACGCACGTACGCGCTGTCTACCGCGTTTTAACCGCCACTAGAAGCGCTTACT
AGTCTCCAGGCACGTGTGAGACCGGCACATGAAAAAAAAAACGTCAGCGGTAC

Strand 2

CGCTGACGTTTTTTTTTTCATGTGCCGGTCTCACACGTGCCTGGAGACTAGTAAGCGCTTCTAGTGGCGGTTAAAA
CGCGGTAGACAGCGCGTACGTGCGTTTAAGCGGTGCTAGAGCTGTCTACGACCAATTGAGCGGCCTCGGCACCG
GGATGCGTTTTTTTTTTCATACTCGAGCATGCTTTTTTTTTTCATGTGCCGGTCTCACACGTGCCTGGAGACTAGTA
AGCGCTTCTAGTGGCGGTTAAAACGCGGTAGACAGCGCGTACGTGCGTTTAAGCGGTGCTAGAGCTGTCTACGA
CCAATTGAGCGGCCTCGGCACCGGGATGCGTTTTTTTTTTCGTCAGCGGTAC

Supplementary Figure S2. DNA sequences of the 338b and 343-353c dinucleosome constructs. The restriction enzyme used to generate the 343-353c cohesive ends is KpnI, while EcoRV for the blunt-ended 338b.

357d

Strand 1

CGCTGCAACGAAAAAAAAAACGCATCCCGGTGCCGAGGCCGCTCAATTGGTCGTAGACAGCTCTAGCACCGCTTA
AACGCACGTACGCGCTGTCTACCGCGTTTTAACCGCCACTAGAAGCGCTTACTAGTCTCCAGGCACGTGTGAGAC
CGGCACATGAAAAAAAAAAGCATGCTCGAGTATGAAAAAAAAAACGCATCCCGGTGCCGAGGCCGCTCAATTGG
TCGTAGACAGCTCTAGCACCGCTTAAACGCACGTACGCGCTGTCTACCGCGTTTTAACCGCCACTAGAAGCGCTTA
CTAGTCTCCAGGCACGTGTGAGACCGGCACATGAAAAAAAAAACGTCCAAGCGGTAC

Strand 2

CGCTTGGACGTTTTTTTTTTCATGTGCCGGTCTCACACGTGCCTGGAGACTAGTAAGCGCTTCTAGTGGCGGTTAA
AACGCGGTAGACAGCGCGTACGTGCGTTTAAGCGGTGCTAGAGCTGTCTACGACCAATTGAGCGGCCTCGGCAC
CGGGATGCGTTTTTTTTTTCATACTCGAGCATGCTTTTTTTTTTCATGTGCCGGTCTCACACGTGCCTGGAGACTAG
TAAGCGCTTCTAGTGGCGGTTAAAACGCGGTAGACAGCGCGTACGTGCGTTTAAGCGGTGCTAGAGCTGTCTAC
GACCAATTGAGCGGCCTCGGCACCGGGATGCGTTTTTTTTTTCGTTGCAGCGGTAC

359d

Strand 1

CGCTGCAAACGAAAAAAAAAACGCATCCCGGTGCCGAGGCCGCTCAATTGGTCGTAGACAGCTCTAGCACCGCTT
AAACGCACGTACGCGCTGTCTACCGCGTTTTAACCGCCACTAGAAGCGCTTACTAGTCTCCAGGCACGTGTGAGA
CCGGCACATGAAAAAAAAAAGCATGCTCGAGTATGAAAAAAAAAACGCATCCCGGTGCCGAGGCCGCTCAATTG
GTCGTAGACAGCTCTAGCACCGCTTAAACGCACGTACGCGCTGTCTACCGCGTTTTAACCGCCACTAGAAGCGCTT
ACTAGTCTCCAGGCACGTGTGAGACCGGCACATGAAAAAAAAAACGTCCAAAGCGGTAC

Strand 2

CGCTTTGGACGTTTTTTTTTTCATGTGCCGGTCTCACACGTGCCTGGAGACTAGTAAGCGCTTCTAGTGGCGGTTA
AAACGCGGTAGACAGCGCGTACGTGCGTTTAAGCGGTGCTAGAGCTGTCTACGACCAATTGAGCGGCCTCGGCA
CCGGGATGCGTTTTTTTTTTCATACTCGAGCATGCTTTTTTTTTTCATGTGCCGGTCTCACACGTGCCTGGAGACTA
GTAAGCGCTTCTAGTGGCGGTTAAAACGCGGTAGACAGCGCGTACGTGCGTTTAAGCGGTGCTAGAGCTGTCTA
CGACCAATTGAGCGGCCTCGGCACCGGGATGCGTTTTTTTTTTCGTTTGCAGCGGTAC

361d

Strand 1

CGCTGCAAAACGAAAAAAAAAACGCATCCCGGTGCCGAGGCCGCTCAATTGGTCGTAGACAGCTCTAGCACCGC
TTAAACGCACGTACGCGCTGTCTACCGCGTTTTAACCGCCACTAGAAGCGCTTACTAGTCTCCAGGCACGTGTGAG
ACCGGCACATGAAAAAAAAAAGCATGCTCGAGTATGAAAAAAAAAACGCATCCCGGTGCCGAGGCCGCTCAATT
GGTCGTAGACAGCTCTAGCACCGCTTAAACGCACGTACGCGCTGTCTACCGCGTTTTAACCGCCACTAGAAGCGC
TTACTAGTCTCCAGGCACGTGTGAGACCGGCACATGAAAAAAAAAACGTCCAAAAGCGGTAC

Strand 2

CGCTTTTGGACGTTTTTTTTTTCATGTGCCGGTCTCACACGTGCCTGGAGACTAGTAAGCGCTTCTAGTGGCGGTT
AAAACGCGGTAGACAGCGCGTACGTGCGTTTAAGCGGTGCTAGAGCTGTCTACGACCAATTGAGCGGCCTCGGC
ACCGGGATGCGTTTTTTTTTTCATACTCGAGCATGCTTTTTTTTTTCATGTGCCGGTCTCACACGTGCCTGGAGACT
AGTAAGCGCTTCTAGTGGCGGTTAAAACGCGGTAGACAGCGCGTACGTGCGTTTAAGCGGTGCTAGAGCTGTCT
ACGACCAATTGAGCGGCCTCGGCACCGGGATGCGTTTTTTTTTTCGTTTTGCAGCGGTAC

351e

Strand 1

CGCTGGAAAAAAAAAACGCATCCCGGTGCCGAGGCCGCTCAATTGGTCGTAGACAGCTCTAGCACCGCTTAAAC
GCACGTACGCGCTGTCTACCGCGTTTTAACCGCCACTAGAAGCGCTTACTAGTCTCCAGGCACGTGTGAGACCGG
CACATGAAAAAAAAAATGCATGCTCGAGTATGAAAAAAAAAATCGCATCCCGGTGCCGAGGCCGCTCAATTGGT
CGTAGACAGCTCTAGCACCGCTTAAACGCACGTACGCGCTGTCTACCGCGTTTTAACCGCCACTAGAAGCGCTTAC
TAGTCTCCAGGCACGTGTGAGACCGGCACATGAAAAAAAAAACCAGCGGTAC

Strand 2

CGCTGGTTTTTTTTTTCATGTGCCGGTCTCACACGTGCCTGGAGACTAGTAAGCGCTTCTAGTGGCGGTTAAAACG
CGGTAGACAGCGCGTACGTGCGTTTAAGCGGTGCTAGAGCTGTCTACGACCAATTGAGCGGCCTCGGCACCGGG
ATGCGATTTTTTTTTTCATACTCGAGCATGCATTTTTTTTTTCATGTGCCGGTCTCACACGTGCCTGGAGACTAGTA
AGCGCTTCTAGTGGCGGTTAAAACGCGGTAGACAGCGCGTACGTGCGTTTAAGCGGTGCTAGAGCTGTCTACGA
CCAATTGAGCGGCCTCGGCACCGGGATGCGTTTTTTTTTTCCAGCGGTAC

353e

Strand 1

CGCTGCGAAAAAAAAAACGCATCCCGGTGCCGAGGCCGCTCAATTGGTCGTAGACAGCTCTAGCACCGCTTAAAC
GCACGTACGCGCTGTCTACCGCGTTTTAACCGCCACTAGAAGCGCTTACTAGTCTCCAGGCACGTGTGAGACCGG
CACATGAAAAAAAAAATGCATGCTCGAGTATGAAAAAAAAAATCGCATCCCGGTGCCGAGGCCGCTCAATTGGT
CGTAGACAGCTCTAGCACCGCTTAAACGCACGTACGCGCTGTCTACCGCGTTTTAACCGCCACTAGAAGCGCTTAC
TAGTCTCCAGGCACGTGTGAGACCGGCACATGAAAAAAAAAACGCAGCGGTAC

Strand 2

CGCTGCGTTTTTTTTTTCATGTGCCGGTCTCACACGTGCCTGGAGACTAGTAAGCGCTTCTAGTGGCGGTTAAAAC
GCGGTAGACAGCGCGTACGTGCGTTTAAGCGGTGCTAGAGCTGTCTACGACCAATTGAGCGGCCTCGGCACCGG
GATGCGATTTTTTTTTTCATACTCGAGCATGCATTTTTTTTTTCATGTGCCGGTCTCACACGTGCCTGGAGACTAGT
AAGCGCTTCTAGTGGCGGTTAAAACGCGGTAGACAGCGCGTACGTGCGTTTAAGCGGTGCTAGAGCTGTCTACG
ACCAATTGAGCGGCCTCGGCACCGGGATGCGTTTTTTTTTTCGCAGCGGTAC

355e

Strand 1

CGCTGACGAAAAAAAAAACGCATCCCGGTGCCGAGGCCGCTCAATTGGTCGTAGACAGCTCTAGCACCGCTTAAA
CGCACGTACGCGCTGTCTACCGCGTTTTAACCGCCACTAGAAGCGCTTACTAGTCTCCAGGCACGTGTGAGACCG
GCACATGAAAAAAAAAATGCATGCTCGAGTATGAAAAAAAAAATCGCATCCCGGTGCCGAGGCCGCTCAATTGG
TCGTAGACAGCTCTAGCACCGCTTAAACGCACGTACGCGCTGTCTACCGCGTTTTAACCGCCACTAGAAGCGCTTA
CTAGTCTCCAGGCACGTGTGAGACCGGCACATGAAAAAAAAAACGTCAGCGGTAC

Strand 2

CGCTGACGTTTTTTTTTTCATGTGCCGGTCTCACACGTGCCTGGAGACTAGTAAGCGCTTCTAGTGGCGGTTAAAA
CGCGGTAGACAGCGCGTACGTGCGTTTAAGCGGTGCTAGAGCTGTCTACGACCAATTGAGCGGCCTCGGCACCG
GGATGCGATTTTTTTTTTCATACTCGAGCATGCATTTTTTTTTTCATGTGCCGGTCTCACACGTGCCTGGAGACTAG
TAAGCGCTTCTAGTGGCGGTTAAAACGCGGTAGACAGCGCGTACGTGCGTTTAAGCGGTGCTAGAGCTGTCTAC
GACCAATTGAGCGGCCTCGGCACCGGGATGCGTTTTTTTTTTCGTCAGCGGTAC

357e

Strand 1

CGCTGCACGAAAAAAAAAACGCATCCCGGTGCCGAGGCCGCTCAATTGGTCGTAGACAGCTCTAGCACCGCTTAA
ACGCACGTACGCGCTGTCTACCGCGTTTTAACCGCCACTAGAAGCGCTTACTAGTCTCCAGGCACGTGTGAGACC
GGCACATGAAAAAAAAAATGCATGCTCGAGTATGAAAAAAAAAATCGCATCCCGGTGCCGAGGCCGCTCAATTG
GTCGTAGACAGCTCTAGCACCGCTTAAACGCACGTACGCGCTGTCTACCGCGTTTTAACCGCCACTAGAAGCGCTT
ACTAGTCTCCAGGCACGTGTGAGACCGGCACATGAAAAAAAAAACGTCCAGCGGTAC

Strand 2

CGCTGGACGTTTTTTTTTTCATGTGCCGGTCTCACACGTGCCTGGAGACTAGTAAGCGCTTCTAGTGGCGGTTAAA
ACGCGGTAGACAGCGCGTACGTGCGTTTAAGCGGTGCTAGAGCTGTCTACGACCAATTGAGCGGCCTCGGCACC
GGGATGCGATTTTTTTTTTCATACTCGAGCATGCATTTTTTTTTTCATGTGCCGGTCTCACACGTGCCTGGAGACTA
GTAAGCGCTTCTAGTGGCGGTTAAAACGCGGTAGACAGCGCGTACGTGCGTTTAAGCGGTGCTAGAGCTGTCTA
CGACCAATTGAGCGGCCTCGGCACCGGGATGCGTTTTTTTTTTCGTGCAGCGGTAC

354f

Strand 1

CGCTGCGAAAAAAAAAACGCATCCCGGTGCCGAGGCCGCTCAATTGGTCGTAGACAGCTCTAGCACCGCTTAAAC
GCACGTACGCGCTGTCTACCGCGTTTTAACCGCCACTAGAAGCGCTTACTAGTCTCCAGGCACGTGTGAGACCGG
CACATGAAAAAAAAAATTGCATGCTCGAGTATGAAAAAAAAAATCGCATCCCGGTGCCGAGGCCGCTCAATTGGT
CGTAGACAGCTCTAGCACCGCTTAAACGCACGTACGCGCTGTCTACCGCGTTTTAACCGCCACTAGAAGCGCTTAC
TAGTCTCCAGGCACGTGTGAGACCGGCACATGAAAAAAAAAACGCAGCGGTAC

Strand 2

CGCTGCGTTTTTTTTTTCATGTGCCGGTCTCACACGTGCCTGGAGACTAGTAAGCGCTTCTAGTGGCGGTTAAAAC
GCGGTAGACAGCGCGTACGTGCGTTTAAGCGGTGCTAGAGCTGTCTACGACCAATTGAGCGGCCTCGGCACCGG
GATGCGATTTTTTTTTTCATACTCGAGCATGCAATTTTTTTTTTCATGTGCCGGTCTCACACGTGCCTGGAGACTAG
TAAGCGCTTCTAGTGGCGGTTAAAACGCGGTAGACAGCGCGTACGTGCGTTTAAGCGGTGCTAGAGCTGTCTAC
GACCAATTGAGCGGCCTCGGCACCGGGATGCGTTTTTTTTTTCGCAGCGGTAC

Supplementary Figure S3. DNA sequences of the 357-361d, 351-357e and 354f dinucleosome constructs. All have cohesive ends generated by KpnI.

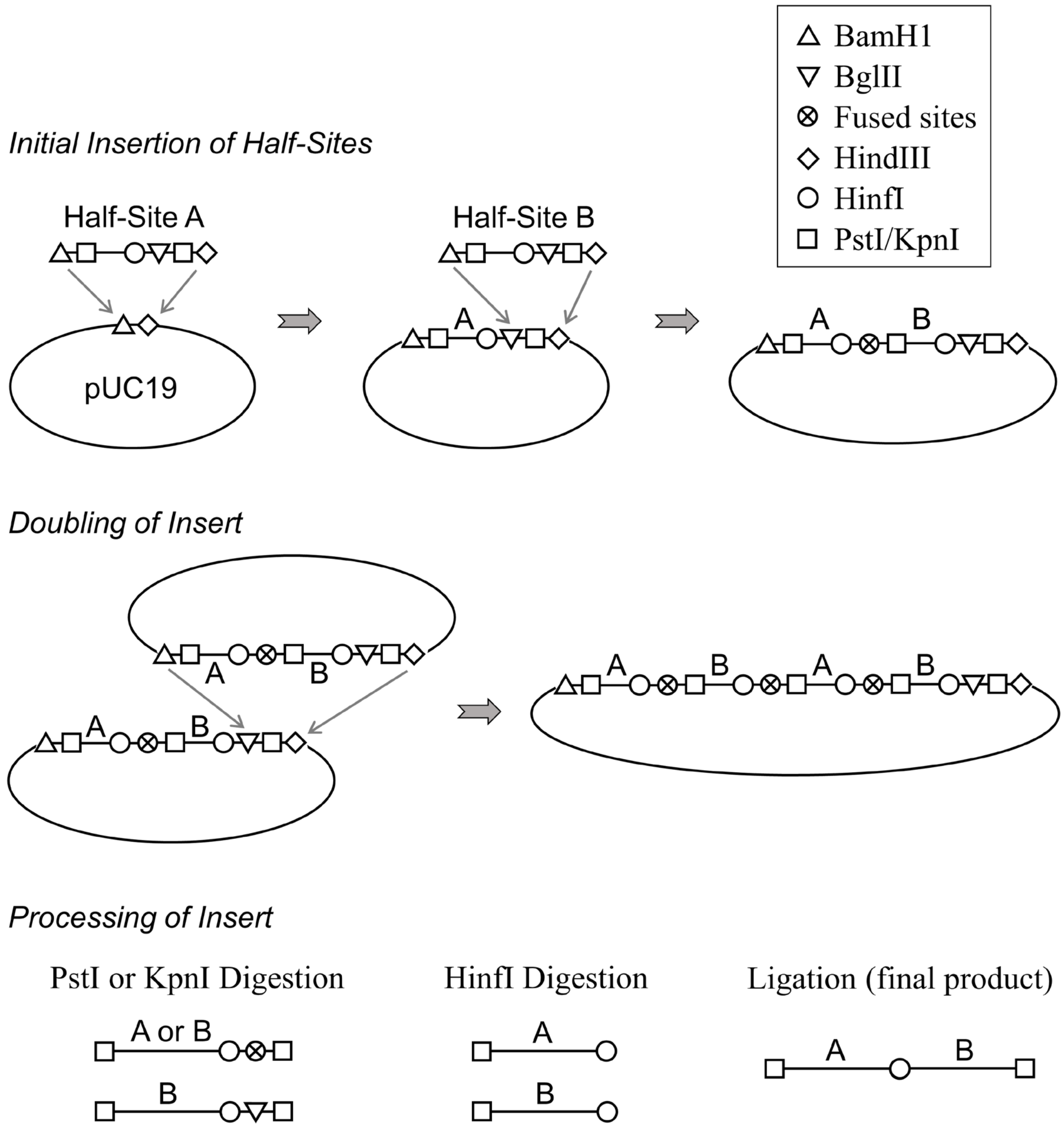

Supplementary Figure S4. Cloning strategy used to produce the palindromic cohesive-ended DNA fragments. Constructs were engineered with either PstI or KpnI termini. The insert doubling approach also applies to the non-palindromic constructs of the a, b and c series, where the insert is flanked by a BamH1 site on one end and BglII + HindIII sites on the other end. Ligation of two HindIII sites on one end leaves this restriction site intact, whereas ligation of BamHI with BglII sites at the other end results in the elimination of these restriction sites (fused sites). For further details, see Materials and Methods.

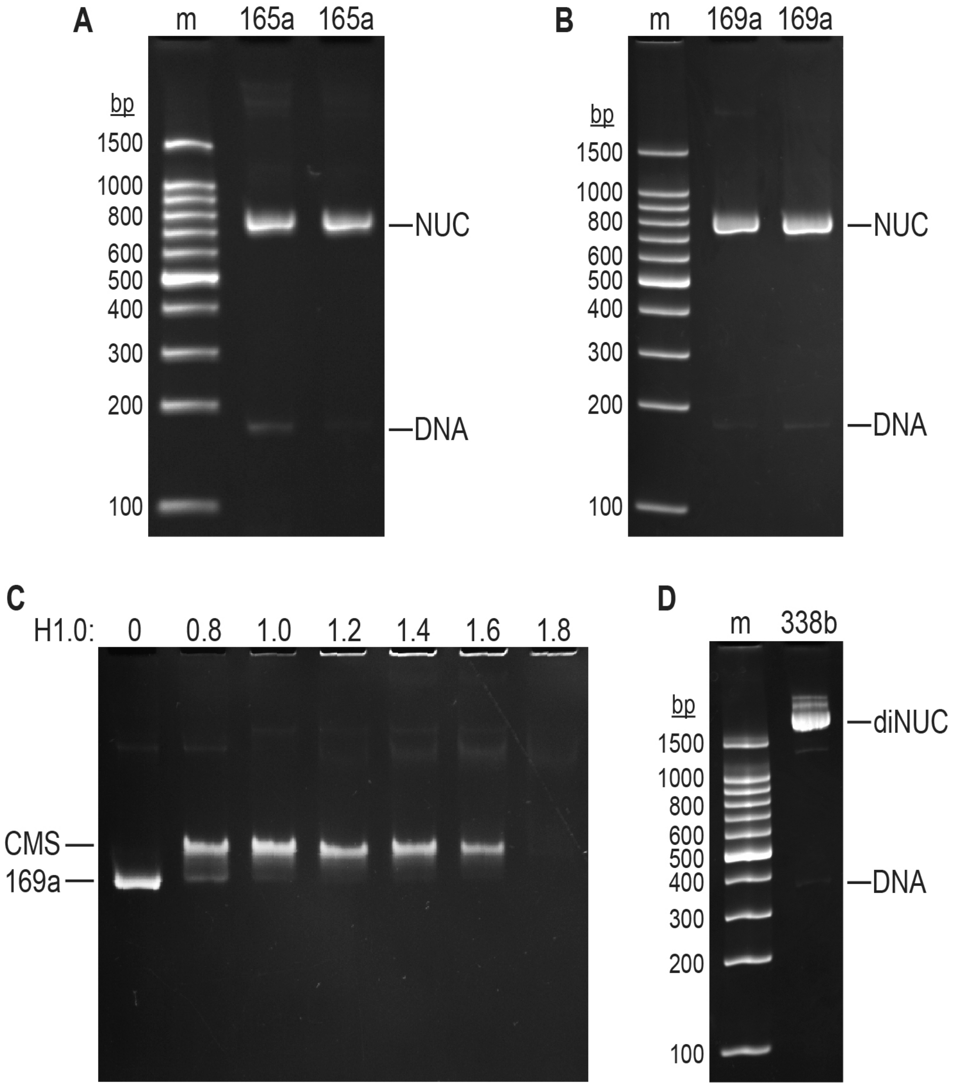

Supplementary Figure S5. Native polyacrylamide gel (6%) electrophoretic analysis of nucleosomal assemblies (a/b series). (**A**, **B**) 165a and 169a mono-nucleosome (NUC) samples from two different reconstitution trials. (**C**) Titration of 169a nucleosome (169a) with H1.0 (H1.0:nucleosome stoichiometry shown), yielding 1:1 nucleosome-linker histone assembly (chromatosome, CMS). (**D**) 338b dinucleosome (diNUC).

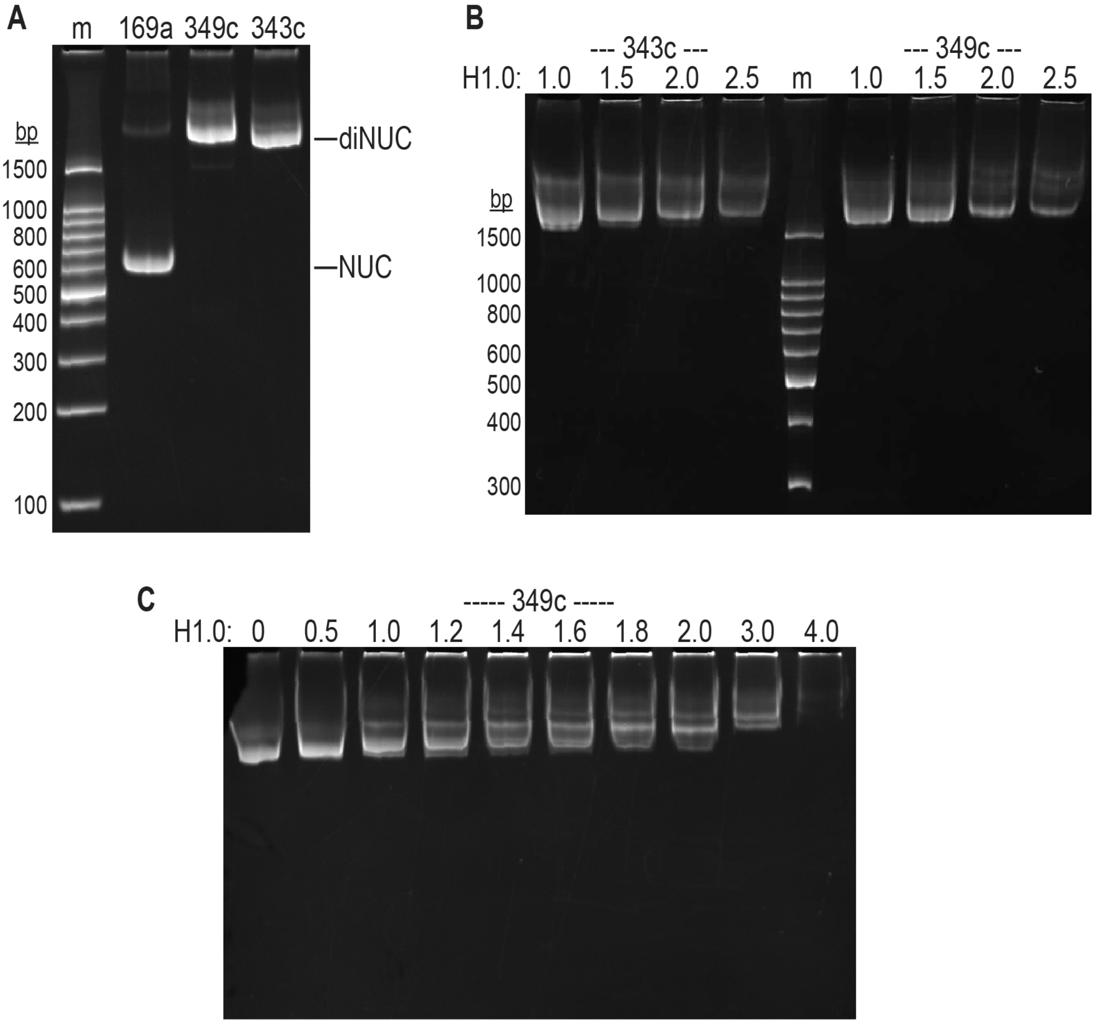

Supplementary Figure S6. Native polyacrylamide gel (6%) electrophoretic analysis of nucleosomal assemblies (c series). (**A**) 343c and 349c dinucleosome (diNUC) in comparison with 169a mono-nucleosome (NUC). (**B**) Assemblies of 343c and 349c dinucleosome with H1.0 (H1.0:dinucleosome stoichiometry shown). (**C**) Titration of 349c dinucleosome with H1x (H1x:dinucleosome stoichiometry shown; sample precipitates upon oversaturation with linker histone).

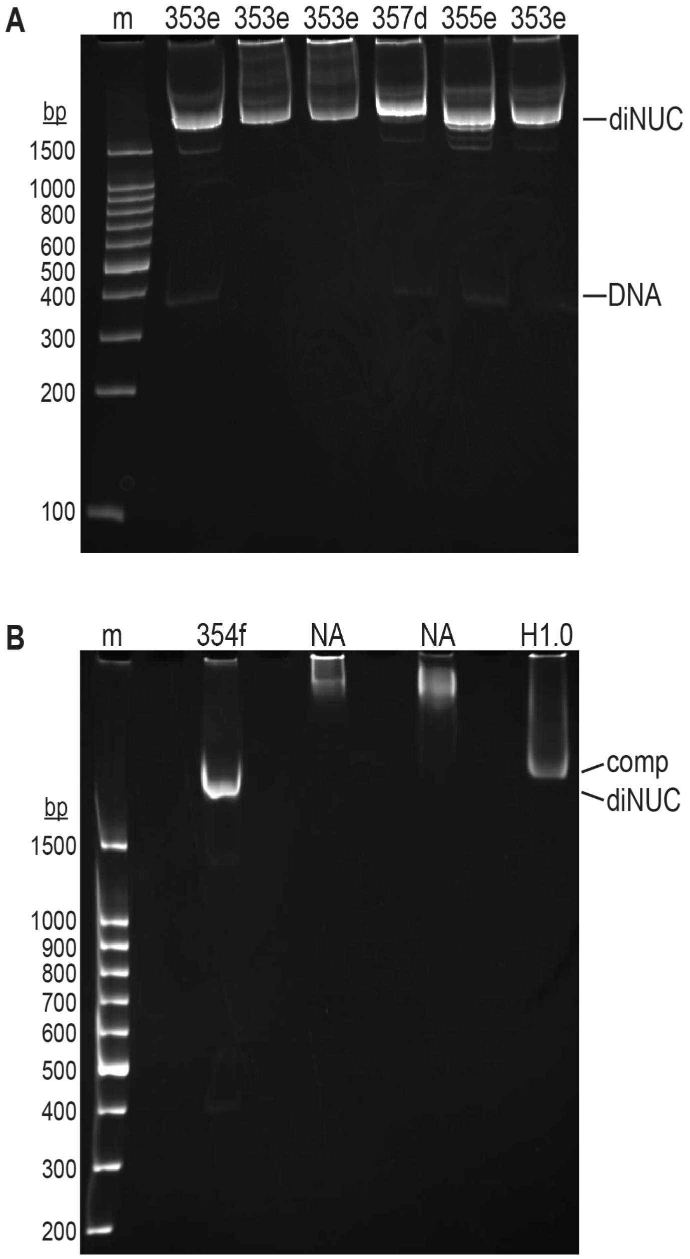

Supplementary Figure S7. Native polyacrylamide gel (6%) electrophoretic analysis of nucleosomal assemblies (d/e/f series). (**A**) 357d, 353e and 355e dinucleosome (diNUC) samples from different reconstitution trials. (**B**) 354f dinucleosome alone (diNUC) and in complex with H1.0 (comp; 2.5:1 H1.0:dinucleosome stoichiometry) or other/additional nuclear factors (NA).

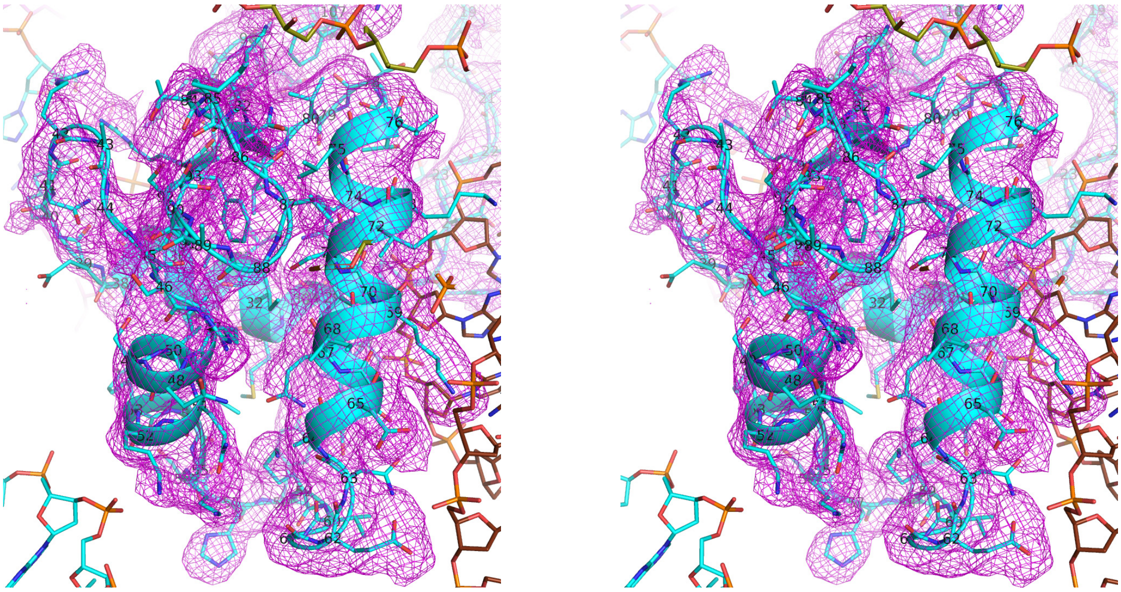

Supplementary Figure S8. Experimental electron density corresponding to linker histone binding in the 169a assembly, in stereo view. An $F_O - F_C$ omit electron density map (magenta; contoured at 1σ; linker histone atoms omitted from the model) in the vicinity of the omitted atoms is superimposed onto the refined model.

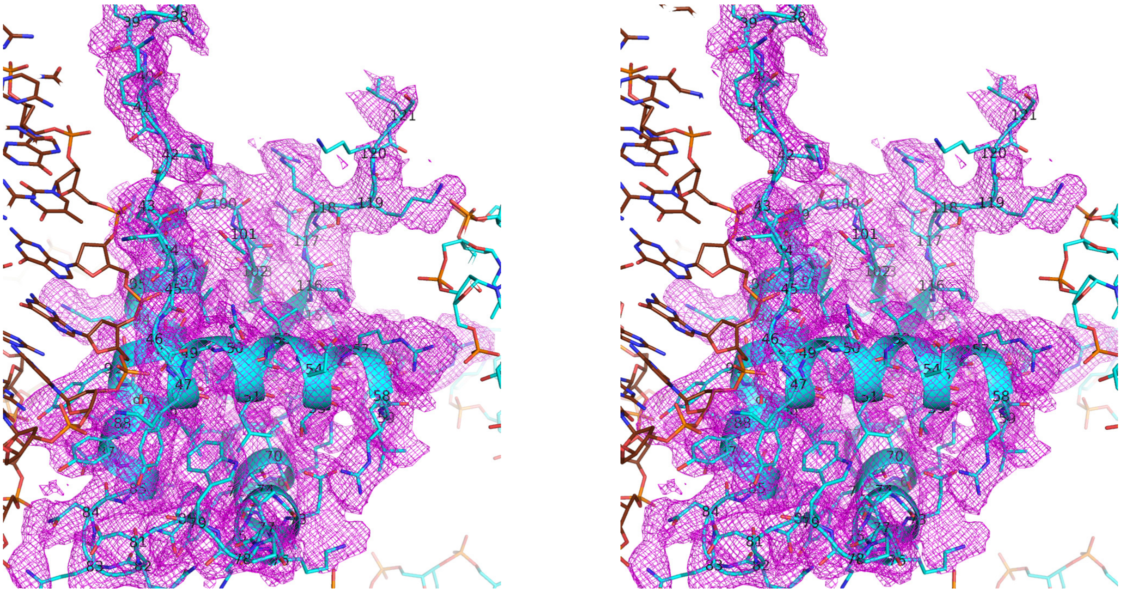

Supplementary Figure S9. Experimental electron density corresponding to linker histone binding in the 338b assembly, in stereo view. An $F_O{-}F_C$ omit electron density map (magenta; contoured at 1.0σ; linker histone atoms omitted from the model) in the vicinity of the omitted atoms is superimposed onto the refined model.

Supplementary Table S1. Data collection statistics for the 353c and 354f dinucleosome crystals

	**353c**	**354f**
**Data collection[a]**		
Space group	$P2_1$	P1
Cell dimensions		
a (Å)	107.50	66.58
b (Å)	203.00	174.32
c (Å)	235.68	214.40
α/β/γ (deg.)	90/94.96/90	87.50/87.80/88.83
Resolution (Å)[b]	5.51–48.91 (5.51–5.81)	5.00–48.25 (5.00–5.22)
Unique reflections	32,479	38,612
R_{merge} (%)	6.7 (165)	24.6 (48.4)
R_{pim} (%)	3.0 (73.9)	24.6 (48.4)
$I / \sigma I$	12.7 (1.1)	2.5 (1.5)
CC½ (%)	100 (46.8)	72.3 (47.4)
Completeness (%)	99.3 (96.7)	92.7 (92.1)
Redundancy	6.8 (6.7)	1.9 (1.9)

[a] Single crystal data.
[a] Data collection wavelength: 1.00 Å (353c), 1.54 Å (354f).
[b] Values in parentheses are for the highest-resolution shell.
